# Side-chain moieties from the N-terminal region of Aβ are Involved in an oligomer-stabilizing network of interactions

**DOI:** 10.1371/journal.pone.0201761

**Published:** 2018-08-06

**Authors:** Kaja Przygońska, Jarosław Poznański, Ulrik H. Mistarz, Kasper D. Rand, Michał Dadlez

**Affiliations:** 1 Institute of Biochemistry and Biophysics, Polish Academy of Sciences, Warsaw, Poland; 2 Department of Pharmacy, University of Copenhagen, Copenhagen, Denmark; 3 Institute of Genetics and Biotechnology, Department of Biology, University of Warsaw, Warsaw, Poland; Rijksuniversiteit Groningen, NETHERLANDS

## Abstract

Oligomeric forms of the Aβ peptide represent the most probable neurotoxic agent in Alzheimer’s disease. The dynamic and heterogeneous character of these oligomers makes their structural characterization by classic methods difficult. Native mass spectrometry, when supported by additional gas phase techniques, like ion mobility separation and hydrogen-deuterium exchange (IM-HDX-MS), enable analysis of different oligomers coexisting in the sample and may provide species-specific structural information for each oligomeric form populated in the gas phase. Here, we have combined these three techniques to obtain insight into the structural properties of oligomers of Aβ1–40 and two variants with scrambled sequences. Gas-phase HDX-MS revealed a sequence-specific engagement of the side-chains of residues located at the N-terminal part of the peptide in a network of oligomer-stabilizing interactions. Oligomer-specific interactions were no longer observed in the case of the fully scrambled sequence. Also, the ability to form alternative structures, observed for WT Aβ peptide, was lost upon scrambling. Our data underscore a role for the N-terminal residues in shaping the equilibria of oligomeric forms. Although the peptide lacking the N-terminal 1–16 residues (p3 peptide) is thought to be benign, the role of the N-terminus has not been sufficiently characterized yet. We speculate that the interaction networks revealed here may be crucial for enabling structural transitions necessary to obtain mature parallel cross-β structures from smaller antiparallel oligomers. We provide a hypothetical molecular model of the trajectory that allows a gradual conversion from antiparallel to parallel oligomers without decomposition of oligomers. Oligomer-defining interactions involving the Aβ peptide N-terminus may be important in production of the neurotoxic forms and thus should not be neglected.

## Introduction

The alarming increase in Alzheimer’s disease (AD) cases in the aging population justifies intense studies on the nature of the causative neurotoxic agent [1]. Studies have led to the widely accepted belief that the main culprit is the aggregation of the Aβ peptide [2,3]. While its monomeric form remains benign [4], the neurotoxicity and synaptotoxicity of even small oligomers have been documented in a number of studies [5,6], drawing attention to soluble oligomers of Aβ peptide, rather than to the amyloid plaques *per se*, as the earliest mediators of neuronal dysfunction [7,8].

Non-monomeric forms of Aβ peptide have become a primary target of structural characterization, as a necessary condition for rational drug design towards AD. However, the details of the evolution of pathological aggregation at the structural level, both on-pathway to fibrils and off-pathway to alternative forms, are still not completely understood. Even the fibrillar state of Aβ peptide proved to be polymorphic [9–11], also when seeded by the brain extracts from different patients [12], leading to the conclusion that the variability extends beyond *in vitro* artifacts. No canonical fibril structure could be established, only the commonalities, like the presence of a “cross-β” structure formed by parallel β-strands, with residues in neighboring chains aligned in strict register. While the precise location of strands is highly variable between structures (see Fig 9 in Ref. [13]), the N-terminal residues retain flexibility in majority of known fibril structures [14].

Oligomers have introduced an additional difficulty. These transient and highly dynamic species naturally coexist in solution [15] as a spectrum of different forms (being either off- or on-pathway to fibrils [16,17]). To apply classic methods of structure analysis, usually a particular form must be at least dominating in the population. Therefore, numerous studies have applied different non-physiological conditions to stabilize a particular form (See also Table A in S1 Appendix with references therein). These studies demonstrated that even small deviations in oligomerization conditions or modifications of the molecule may lead to an astounding variety of structurally diverse forms. Without knowledge of the structures populated under native conditions, it is difficult to place this variety of forms upon the landscape of native/non-native and on/off-pathway forms. Therefore, more attention has been directed toward the characterization of the forms obtained in, or close to native conditions, without modifications and stabilizing agents of any kind. Solid state NMR measurements of such samples commonly identified the presence of β-structure content, which increased over time in the course of the evolution of oligomeric forms [17–19]. A classic solution-phase HDX-MS approach carried out for freshly dissolved samples of Aβ 1–40 [20–22] identified an equilibrium of monomers of unrestricted exchange and LMW oligomers that were partially protected. Interestingly, also a common feature of these studies [18,19,23], supported by IR [24], was a transient formation of an antiparallel β-structure, persistent up to the stage of protofibrils and followed by the change to an in-register parallel β-structure [18]. The presence of an antiparallel arrangement of chains was also common among stabilized oligomers (See also Table A in S1 Appendix). In general, structural studies led to the hypothesis that early stage oligomers consist of stacked antiparallel β-hairpins [19,25] which are further converted into parallel β-sheets. Such a conversion assumes the concerted structural transformation, in result of which antiparallel intramolecular H-bonds, specific for β-hairpins, are replaced by intermolecular parallel H-bonds, characteristic for fibrils. However, the detailed mechanistic model that would allow chain rotation without decomposition of an oligomer has not been proposed yet.

In the majority of cases, structural studies have focused on the C-terminal part of Aβ peptide (17-40/42) which provides the core of the amyloid, whereas the role of the N-terminal region remains less clear. Nearly all hydrophobic residues with β-structure propensities map to the C-terminal part. On the other hand, a peptide variant 17-40/42, deprived of 16 N-terminal residues, is a natural product (p3 peptide) of the alternative pathway of amyloid precursor protein cleavage, called “non-amyloidogenic” [26]. Thus, p3, resulting from “non-amyloidogenic” cleavages, contains all “amyloidogenic” properties and all determinants required for fibril assembly. Neurotoxic properties, believed to result from oligomerization, should thus also be encoded solely within the p3 product [27]. On the other hand, neurotoxic forms of Aβ peptide are expected to be the result of the amyloidogenic pathway only, and not arising from the non-amyloidogenic pathway. For this reason p3 is believed to be non-neurotoxic [28], though its role was not studied in much detail [29]. If p3 is non-neurotoxic, while longer 1-40/42 variants are, then the presence of the N-terminus is what makes this peptide pathogenic. N-terminus may affect neurotoxicity in different ways [30], and its impact at each level, including oligomerization step, needs to be studied.

In summary, the knowledge on evolving oligomeric structures is still not satisfactory, and no consensus on their molecular models has so far been obtained. Moreover, standard methods provide information only on the average properties of an ensemble while co-existing minor forms, a possible source of neurotoxicity, may remain unobserved. Analysis of minor species was made possible by use of NMR spectral filter [31] or ^19^F NMR [32], indicating immediate formation of a class of β-sheet-containing oligomers after dissolution.

Structural studies of the assembly process of Aβ peptide may also benefit from alternative approaches that would provide a more species-specific structural information and characterize better the complete spectrum of forms co-evolving in solution. Native mass spectrometry (native MS) provides such an alternative since it allows to resolve signals originating from species of different non-covalently stabilized forms that coexist in solution [33], for instance oligomers of different orders or their alternative structural forms. Inside the mass spectrometer, gaseous protein ions can be probed by a panel of gas phase techniques [34], like ion mobility (IM) [35] that additionally resolves species according to their collisional cross section (Ω, Å^2^) or gas-phase HDX [36,37] which probes the involvement of side-chain protons in intra- or intermolecular H-bonding. MS-based methodology can thus also provide structural information for each of the protein species detected separately. Such a unique insight, not accessible with other methods, justifies application of MS-based methods to structural studies of oligomerizing species, despite the necessity to transfer the oligomers from solution to the gas phase. It has been shown that solution-like conformational states can be maintained in the gas phase for tens to several hundreds of milliseconds after gentle ionization [38–40]. Gas phase techniques applied in this time-frame, such as IMS and gas-phase HDX-MS, can thus provide information on solution-like structures. Still it has to be taken into account that structures observed in the gas phase may differ from solution structures to a degree difficult to assess. IM-MS has recently been applied in the studies of a variety of oligomerizing molecules [41,42], including Aβ peptide and its variants [43,44]. These studies revealed a spectrum of Aβ oligomers of different orders and their alternative structural variants. A linear increase in collisional cross-section with the number of Aβ peptide units has been observed, indicating a planar growth of the oligomer in 2D rather than globular growth in 3D [43].

**Fig 1:**
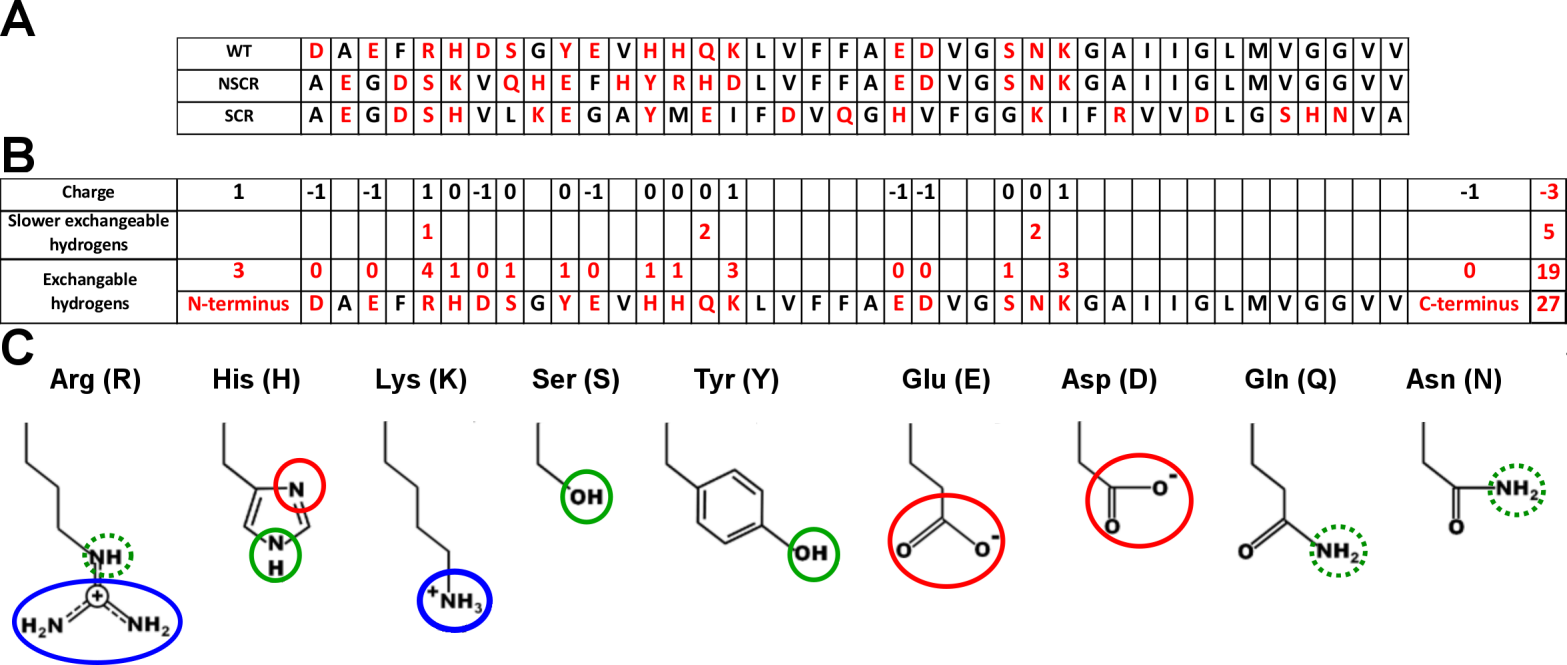
(A) Sequences of WT Aβ 1–40 and its two variants with scrambled N-terminal 1–16 amino acids (NSCR) and with all amino acids scrambled (SCR). Protonation sites on side-chain moieties are highlighted in red. (B) Sequence of WT Aβ 1–40 with number of exchangeable hydrogens and charges. (C) Exchangeable side-chain hydrogens present in neutral forms of residues of Aβ 1–40. Protonation sites on basic side-chain marked in blue, neutral side-chain in green. Proton accepting groups are highlighted in red. Slower exchangeable side-chain hydrogens marked in dotted green.

Here, we applied IM and gas-phase HDX-MS for the analysis of freshly prepared Aβ 1–40 to study the spectrum of oligomeric forms co-existing in the sample. Gas-phase HDX-MS reports on the exchange of non-amide heteroatom-bound protons, while backbone amide protons observed in classic solution HDX-MS exchange too slowly in the gas phase to be observed [37,45,46]. Interestingly, exchangeable side-chain protons in Aβ peptide are localized nearly exclusively in the N-terminal region (mainly between residues 1 and 16 –Fig 1). Thus, this approach could be particularly sensitive to examine the involvement of N-terminal region side-chain protons in oligomer-stabilizing network of interactions.

## Materials and methods

### Materials

The Aβ peptide was obtained by expression in *Escherichia coli* and purified by HPLC as described previously [47]. The vectors of Aβ 1–40 scrambled sequence (SCR) and Aβ 1–40 scrambled sequence 1–16 (NSCR) in pET-30a(+) were purchased from GenScript and the peptides were expressed in *Escherichia coli* and then purified by HPLC as described previously [47]. The identity and purity of all peptides were verified using a Q-ToF Premier ESI-MS instrument (Waters Corp., Wilmslow, U.K.). Typically, the concentration of the peptide in the stock solution was 100 *μ*M. NaOH, HCl, Tris, and 26% ammonia-D_3_ in D_2_O 99.5% (ND_3_/D_2_O) was purchased from Merck (Darmstadt, Germany) and ammonium acetate from Fluka (The Netherlands).

### Ion mobility mass spectrometry

Experiments were performed using a hybrid Q-TOF mass spectrometer with IM capabilities—Synapt G2 HDMS (Waters Corp. Wilmslow, UK). Samples of Aβ 1–40 WT, SCR, NSCR at 100 *μ*M concentration in 10 mM CH_3_COONH_4_, pH 7.4 (when necessary, pH was adjusted with ammonia) were infused directly (at 7 *μ*L/min) to the ion source of a mass spectrometer, with a glass Hamilton syringe, through a stainless steel capillary. The mass signals were measured in the range 400–4000 *m/z* at the rate of 1 scan per second. The spectra analyzed were the average of 200 scans. The instrument was tuned to obtain the best possible signal and HDX efficiency, using the electrospray positive ion mode with a capillary voltage of 2.8 kV and a sample cone voltage of 37 V. The source and desolvation temperatures were maintained at 85 and 180°C, respectively. The mobility T-wave cell was operated at a pressure of 2.5 mbar of nitrogen, with a wave velocity of 300 m/s and amplitude (T-wave height) of 40 V. Data acquisition and processing were carried out with MassLynx V4.1 (Waters) and DriftScope V2.1 (Waters) software supplied with the instrument. Each analysis of drift time profiles in Aβ WT, SCR, NSCR was carried out under the same experimental conditions. All data were repeated for batch-to-batch replicates (n = 3 or more) to confirm the reproducibility of the results.

### Gas-phase HDX-MS

Gas-phase HDX-MS was performed in the ion source region of a commercially available Synapt G2 HDMS instrument immediately downstream of the primary cone exit (sample cone) as described elsewhere [45]. In short, 2.0 ml of aqueous ND_3_/D_2_O reagent was added to the standard ETD reagent vial, with the ETD reagent removed. To control reagent flow, the mass spectrometer software was set in ETD-mode with a HDX reagent gas flow rate of 0–50 mL/min (N_2_ gas), 50 mL/min is the maximum setting on the Synapt G2 HDMS instrument. N_2_ gas was passed through the headspace of the reagent pot and through a needle (corona discharge needle), downstream of the primary cone exit. The trap T-Wave wave-height was set to +6.0 V to prevent unwanted electron transfer reactions in case of residual ETD reagent in the tubing. Control experiments were additionally performed under identical conditions with the only difference being the presence of undeuterated NH_3_/H_2_O, instead of ND_3_. This procedure allowed us to test if the presence of a basic gas would affect the experiment, and no changes in either ionization, adduct formation, or conformation were observed.

### Data analysis

Processing of all mass spectra was carried out using MassLynx V4.1 software with a Savitzky-Golay smoothing function (3, 5) and subsequent centering of the peaks. The deuterium content of peptides was determined using Excel 2013 (Microsoft Corp., Redmond, WA, USA) by calculating the difference in the intensity weighted centroid average masses of deuterated ions with respect to those from a non-deuterated control sample recorded in the absence ND_3_ gas. All data shown derive from at least three or more replicate measurements. The error bars shown in the figures represent the standard deviation (SD) of such replicate measurements.

For deconvolution of the complex, split isotopic envelopes, corresponding to the differently exchanging species, an in-house procedure was developed. First, the procedure allowed us to calculate mass distributions expected at each stage of exchange for a single uniform conformation for a peptide of a given mass and number of exchangeable protons. The calculation was carried out using a simplifying assumption of equal probability of exchange for each exchangeable proton. If the probability is not equal (which is highly likely), the distributions necessarily become narrower. So, the result of this assumption is that we can model the widest possible distribution expected for a single state. As a result, the number of detected states may only be higher, but cannot be lower. Using this approach, we thus identified the minimum number of states present during exchange that would account for the experimental distributions. As a result of the first step a set of 200 theoretical uniformly deuterated single-state distributions ranging from 0% to 100% deuterium uptake was simulated for each sample. For the deconvolution procedure, both centrioided experimental distributions and theoretical distributions were represented as 500-element vectors, calculated by convolution with a gaussian function and sampling at points uniformly distributed along the specified mass range of theoretical isotopic envelopes. The resulting linear equations were solved using boosted Gold algorithm as described in Ref. [48] with 10000 iterations, 100 boosting steps and p = 1.2. This allowed us to obtain a linear combination of single-state distributions that fits the measured distribution while meeting physical constraints (small number of non-zero elements, no negative elements). In case of two neighboring non-zero elements in the solution vector, only a single component distribution was reported in the results, with deuterium uptake linearly interpolated between theoretical uptakes corresponding to these elements.

### Circular dichroism spectroscopy

For CD measurements, Aβ WT and SCR samples were prepared as described above, at 100 *μ*M concentration in 10 mM CH_3_COONH_4_, pH 7.4 (when necessary, pH was adjusted with ammonia). CD spectra were recorded at wavelengths from 270 to 210 nm using a J-815 CD spectrometer (Jasco, Halifax, Canada). The molar ellipticity was calculated according to the formula [θ] = θ/(c·l), where θ is the measured ellipticity in millidegrees; c is molar peptide concentration; l is the optical path length of the cuvette in millimeters.

### Dynamic light scattering

All DLS experiments were carried out at 25°C with a DynaPro NanoStar 192-DPN apparatus (Wyatt Technology, Santa Barbara, CA, USA) equipped with a 661 nm laser. The autocorrelation function (ACF) for the light scattered by Aβ WT and SCR (at 100 *μ*M concentration in 10 mM CH_3_COONH_4_, pH 7.4) solution placed in an Eppendorf UVette disposable cuvette (50–2000 *μ*l) was measured and further analyzed in the range of 0.5 *μ*s to 0.2 s using Dynamics software (Wyatt Technology, ver. 7.0.2.7). All samples were filtered with 0.45 *μ*m pore size syringe filter and additionally centrifuged (9000 *g*) for 3 minutes directly before the measurement. For each sample, a series of at least 3 successive repetitions, 50 acquisitions of 10 s in each, were collected, and those 10 s accumulations with abnormally high SOS function and/or with highly fluctuating SLS signals were removed from further analysis. Since no apparent time trend in SLS data was observed, the DLS data collected upon the first repetition (i.e., during the initial 10 min after sample dilution) were averaged and further analyzed.

### Modeling of WT Aβ 1–40 oligomers

All the molecular modeling and MD simulations were performed using YASARA-Structure Ver. 17.8.15 [49]. The initial protofibril-like structure of a non-covalently stabilized hexamer of WT Aβ was prepared as described previously [43], using constraints deduced from accessible fibril structural information: intermolecular D23-K28 salt bridge, residues L17-V24 and A30-V39 kept in the extended conformation to build a β-sheet core, the twist of the backbone in the turn region and the intrachain distance constraints (2.9–8.5 Å between C^α^ atoms) set in agreement with mutational data for F19-G38 and A21-V36 residue pairs to simulate their spatial proximity [50].

The initial hairpin-like structure of WT Aβ was prepared using constraints adopted from Aβ 1–40 complex with antibody protein Z_Aβ3_ (PDB ID: 2OTK [25]). This included a pattern of H-bonds (distance constraints for appropriate N …O pairs) and a weak dihedral (compatible with the antiparallel β-sheet backbone) and pseudo-dihedral constraints that kept the proper geometry of interacting centers.

The formation of putative geometries of various oligomeric forms was forced by additional constraints mimicking formation of the assumed set of intermolecular hydrogen bonds (N…O distance, backbone geometry, relative orientation of C = O and H-N centers). All these transient states were rationally selected to avoid the global unfolding of the large parts of the studied systems, so only local changes were analyzed using a simulated annealing method.

Putative pathways of oligomer inter-conversions were analyzed with the aid of a constraint-driven simulated annealing strategy. The weights of constraints were initially modified to stimulate the system to adopt the required geometry, and further decreased while the required structure was "induced". MD simulations for putative transition states were performed in the isothermal-isobaric (NTP) ensemble (T = 298 K and p = 1 atm). The model of WT Aβ 1–40 dimer (2 hairpins, Figure H, panels i-iv in S1 Appendix), which was found to be virtually stable, was further subjected to the 20 ns unconstrained MD. Details of constraints and weighs were described in Text A in S1 Appendix.

It should be noted that the presented molecular trajectory is an MD verification at the atomic level of a hypothetical, speculative scheme showed in Discussion section. However, our data demonstrate a consistent pathway of successive conversion of an ensemble of antiparallel β-hairpin-like monomers to the parallel β-sheet sandwich oligomer, for which no global decomposition to monomers is required.

## Results

### Gas-phase HDX-MS of WT Aβ 1–40 oligomers

Native MS was performed at room temperature, pH 7.4, on freshly prepared samples of Aβ 1–40 and its two variants in which the sequence of amino acids was scrambled, either along the entire sequence (SCR) or along its N-terminal 16 amino acids (NSCR). Sequences of all peptides are shown in Fig 1. Gas-phase HDX-MS experiments were carried out on the oligomeric forms of these peptides in the cone-exit region of the mass spectrometer, using a setup described in more detail elsewhere [45]. Gas-phase HDX using ND_3_ gas, executed at short timescales after ESI, can report on conformational differences between different structural forms of peptides and proteins [36,37,45,51]. In contrast to solution-phase HDX-MS, where the labeling of backbone amide hydrogens occurs across seconds to several hours, millisecond timescale gas-phase HDX-MS reports more directly on structure, with less influence from dynamics and flexibility. Conformational changes occur at much slower rates in a vacuum because of the absence of solvation that lowers the energy required for conformational rearrangements [45,46,52].

**Fig 2:**
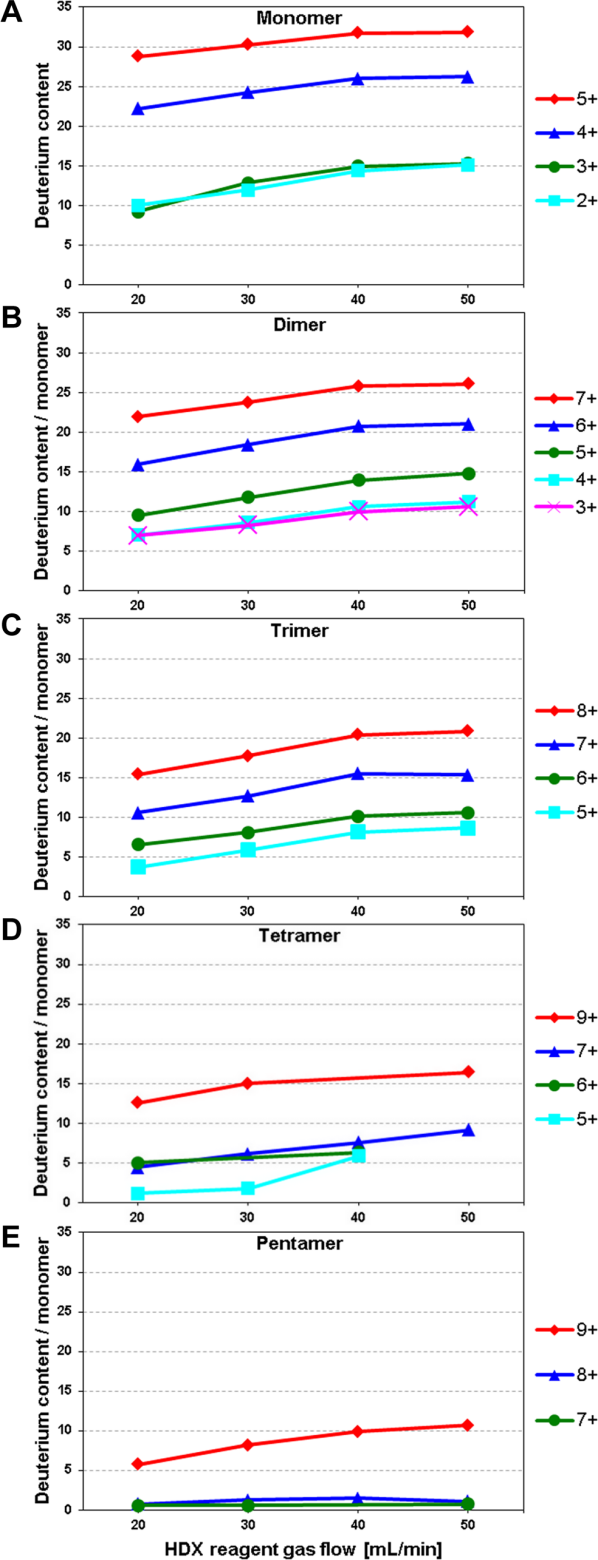
Deuterium content per monomer (HDX/M) of individual charge states of monomers and oligomers of WT Aβ 1–40, induced by gas-phase HDX-MS as a function of make-up gas flow rate (20–50 mL/min) averaged over all signals of the appropriate isotopic envelope.

Native MS spectra of Aβ 1–40 contain numerous signals corresponding to different oligomeric forms (Figure A in S1 Appendix). These signals were previously assigned to specific oligomers and their charge states [43]. Here, the isotopic envelopes for low order oligomeric forms were analyzed before and after exchange, monitored at four HDX reagent flow values: 20, 30, 40, and 50 mL/min. At lower HDX reagent flow rates, deuterium labeling was lower, as observed before [45,46], while higher rates led to a decreased signal intensity. As the first step of analysis, the average deuterium uptake (calculated from the average peptide mass after exchange) was measured. In Fig 2, the average deuterium uptake for monomers to pentamers is shown, normalized per monomeric unit. The uptake was strongly dependent on the charge of the ion, as expected. For monomers (Fig 2A), deuterium uptake of 32 D was observed for charge state 5+ (MON^5+^). The neutral form of Aβ 1–40 molecule contains 27 non-amide heteroatom-bound protons (listed in Fig 1) that would be expected to undergo exchange during gas-phase HDX-MS, along with charging protons. MON^5+^ contained five such charge-carrying exchangeable protons, giving a total of 32 exchangeable protons. MON^5+^ exhibited complete exchange (32 D). For MON^4+^, the exchange was 20% lower than for MON^5+^. At still lower charges the exchange level decreased accordingly, to approx. 15 D for charges 2+ and 3+. The collisional cross-section Ω value, measured in a previous study [43], decreased from 770 Å^2^ for MON^5+^ form to 600 Å^2^ for MON^2+^, indicating only a minor structural collapse with decreasing charge. Our current data did not allow us to estimate whether such a change in collisional cross-section could explain the 2-fold decrease in HDX. It is also likely that more exchange-competent complexes were formed between ND_3_ and molecules of higher charge than lower charge. A comparison of HDX levels of differently charged polypeptide ions should thus be approached with caution, as also explained elsewhere [46,51].

For the dimeric species (Fig 2B) a similar strong charge dependence was observed. At the highest charge state of 7+, the deuterium exchange per monomer (HDX/M) was 26 D, which was comparable to the exchange observed for MON^4+^ (26 D). For these two states the charge density (charge/number of monomers in oligomer) was comparable, being 4 for the monomer and 3.5 for the dimer. In this case, the level of exchange correlated roughly with the charge density per monomer. With decreasing charge, the exchange in the dimer was significantly decreased, to 21 D for 6+ and to 10 D for 3+. The accompanying differences in the collisional cross section of the dimer were small (Ω = 966 Å^2^ for 3+ vs. 952 Å^2^ for 5+). The dependence on charge was retained for higher order oligomers–trimers to pentamers (Fig 2C–2E), with HDX/M values becoming much smaller, as low as 1–2 D for PEN^7+^. In case of the trimeric TRI^6+^ signal, two alternative states were detected, of different Ω values: 1269 Å^2^ and 1533 Å^2^ (see Table 1 in Ref. [43]). These two distinct states of different Ω are also characterized by different deuterium uptake values (see below).

**Fig 3:**
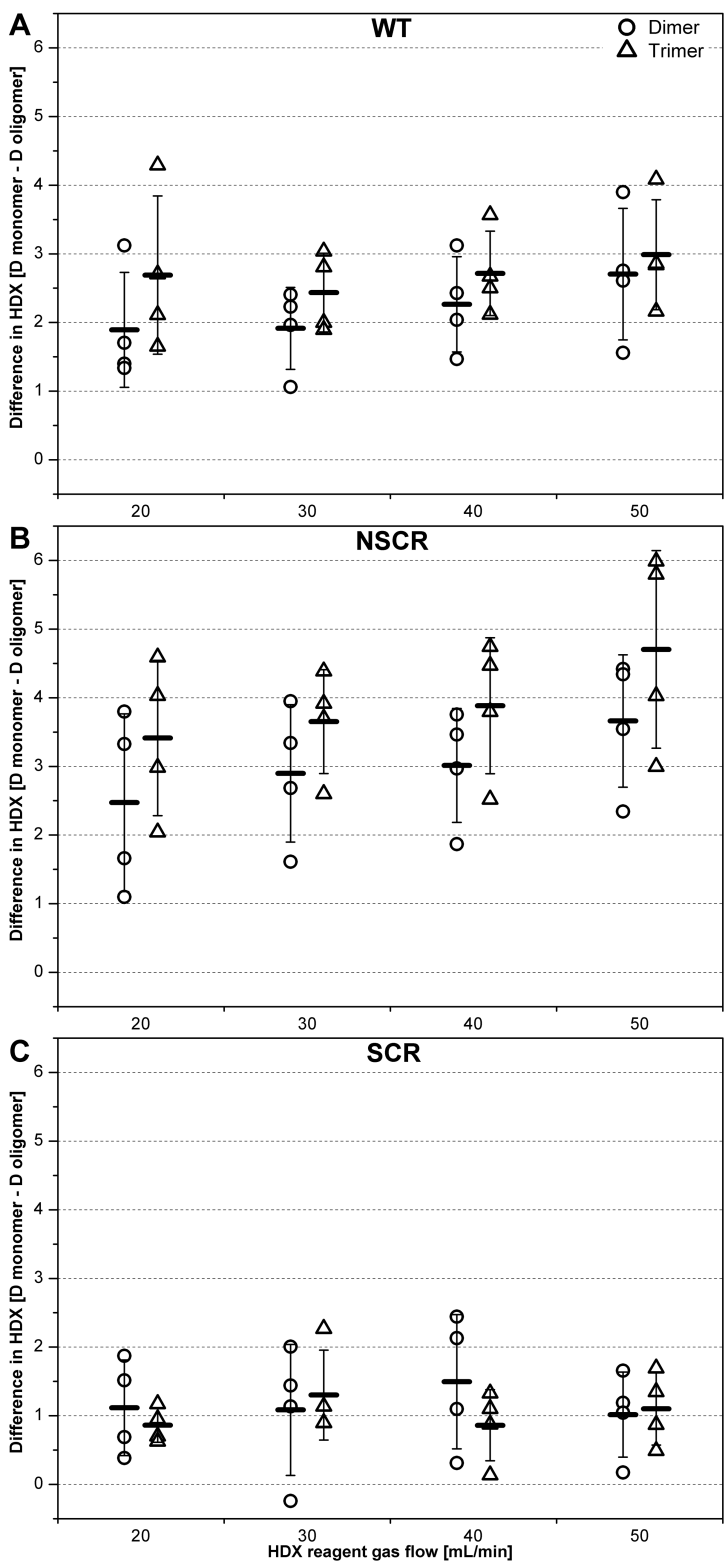
Difference in deuterium uptake per monomer between dimers and monomers (circles) and trimers and monomers (tringles) in WT Aβ 1–40 (A), NSCR Aβ 1–40 (B) and SCR Aβ 1–40 (C), based on the analysis of the m/z region 2164–2169 which contains well resolved signals from MON^2+^, DIM^4+^, TRI^6+^ oligomeric forms bearing the same charge per monomer. The difference in deuterium uptake (D) is shown at the vertical axis and HDX reagent gas flow rate (20–50 mL/min) at the horizontal axis. Four batch-to-batch replicates are represented on the scatter. Dashes refer to the mean difference. Error bars indicate the standard deviation calculated from replicate measurements (number of replicates n was 4).

Due to the strong dependence on charge density, exchange levels between different oligomeric states can be directly compared only for oligomer forms bearing the same charge per monomer. For instance, the isotopic envelope in the *m/z* region 2164–2169 contained well resolved signals from MON^2+^, DIM^4+^, TRI^6+^ (compact and extended forms) and TET^8+^ (see also Figure A, panel i in S1 Appendix and Fig 2 in Ref. [43]), characterized by the same charge density of 2 per monomer. The difference in uptake in dimers and trimers (compact form), as compared to monomers was thus calculated for these species as an average over several experiments (Fig 3A). This analysis showed that uptake in dimers and trimers was on average smaller by 2–3 D than in monomeric forms. Thus, at least two more exchangeable protons were on average involved in the interaction network stabilizing oligomeric forms, as compared to monomers.

**Fig 4:**
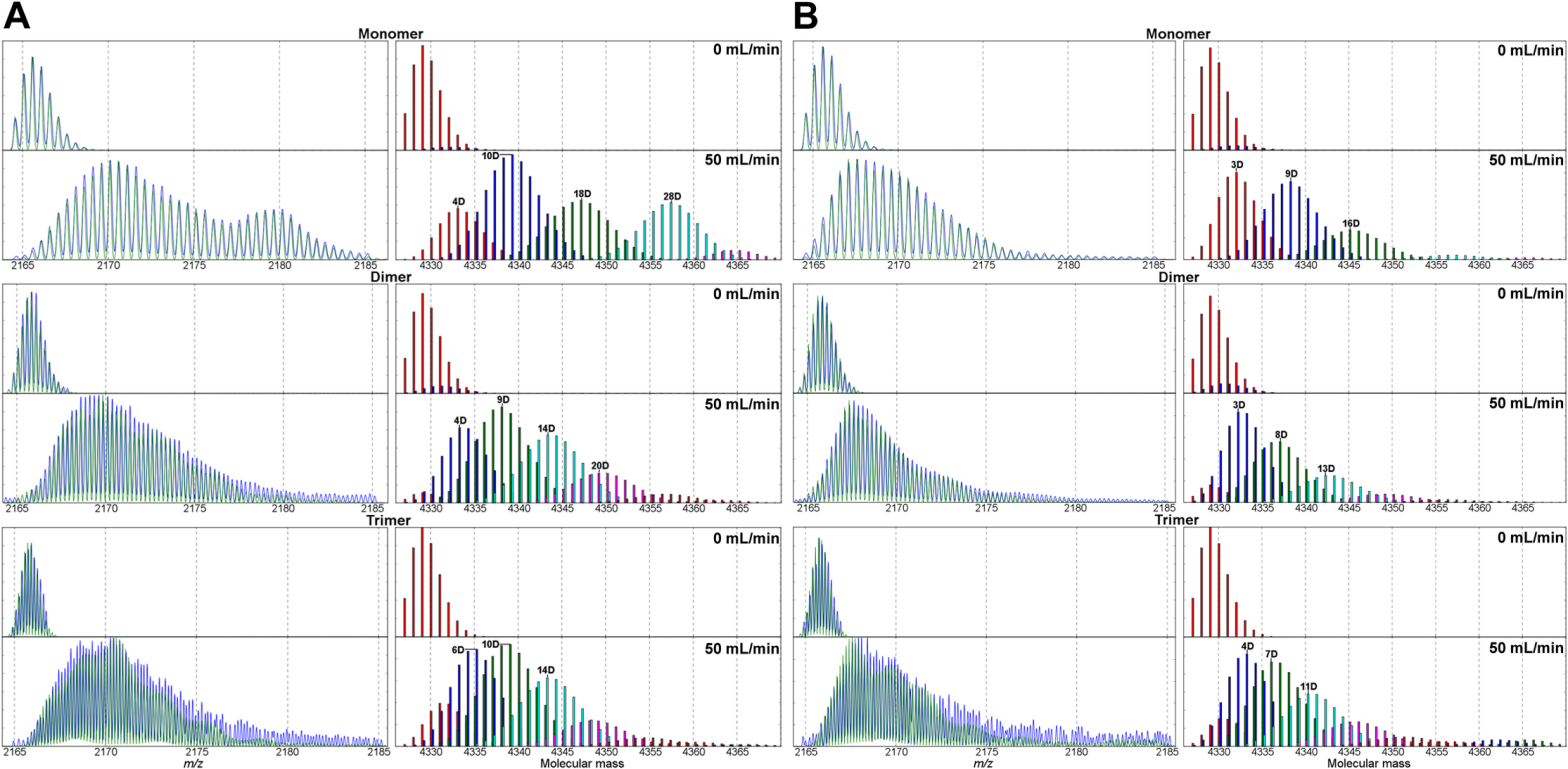
Analysis of the isotopic envelopes after gas-phase HDX-MS for selected signals of species bearing the same charge per monomer, namely MON^2+^, DIM^4+^ and TRI^6+^ (2164–2185 m/z range) of WT Aβ 1–40 (A) and SCR Aβ 1–40 (B). Left panels—isotopic envelopes corresponding to MON^2+^ (upper panels), DIM^4+^ (middle panels), TRI^6+^ (lower panels), for make-up gas flow 50 mL/min in the presence of ND_3_/D_2_O reagent. Right panels—decomposition of the experimental isotopic envelope into a family of isotopic envelopes of FWHM, expected for a single conformational state. The spectra were recalculated from *m/z* domain, (shown in left panels) to the domain of molecular mass of a monomeric unit in an oligomer (shown in right panels). Results of replicates of this experiments are shown in Figure C in S1 Appendix.

A more detailed inspection of the deuterium exchanged signals of the monomer, dimer and trimer in the *m/z* region 2165–2185 (Fig 4A, left panels, Figure C, panels i-iii in S1 Appendix) revealed that the isotopic envelopes were strongly split into several forms, exchanging with different efficiency. For gas-phase HDX-MS experiments carried out using the same experimental setup on smaller, largely unstructured peptides, like Leu-Enk and Glu-fibrinopeptide B (Figure B, panel i in S1 Appendix), no such widening of the isotopic envelope following exchange was observed, indicating that such peptides exist as a more homogeneous ion population of similar structures. However, similar control experiments on the model protein cytochrome c (Figure B, panel ii in S1 Appendix), showed that the full width at half maximum (FWHM) (30 D, 8+ charge, 12.4 kDa) of the isotopic envelope after exchange was also wider than expected for a single conformer, indicating the existence of several differently exchanging species of this larger protein. Similarly large FWHM values were observed in other studies of proteins [36,45]. Thus, for the much smaller Aβ monomers (4.3 kDa), dimers (8.6 kDa), and trimers (12.9 kDa), the overall FWHM was thus relatively very wide (25 D) and in fact clearly split into several distinct species, as the envelopes were not following a single uniform Gaussian distribution. The isotopic envelope distributions at different stages of exchange expected for a single, uniform conformation can be calculated and used to establish the family of forms best explaining the observed wide distribution of masses. For this aim, an in-house developed software was used to fit the family of uniform, single-state distributions to the experimental set of signals before and after exchange, as described to more detail in Methods section.

**Fig 5:**
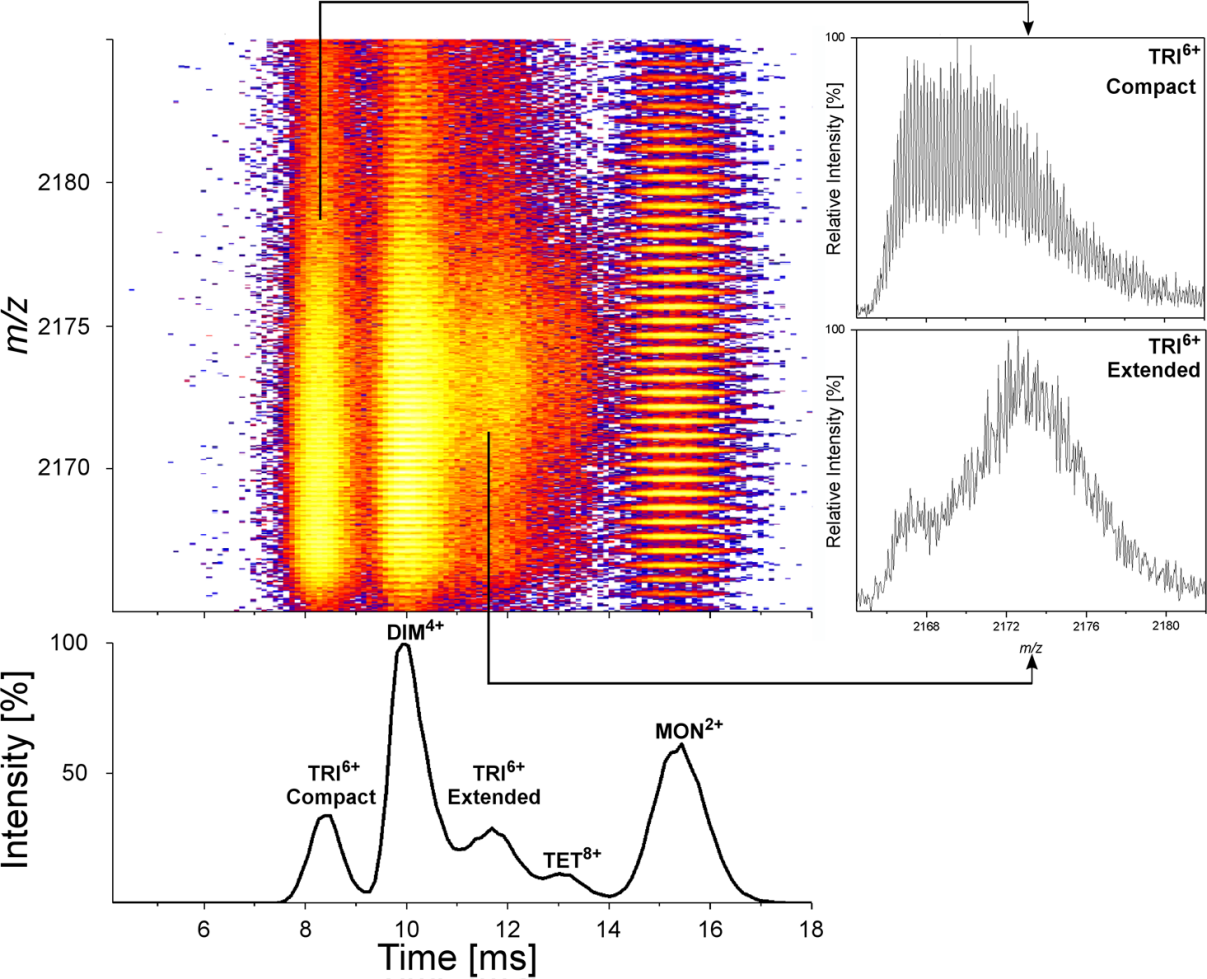
Enlarged fragment of a two dimensional IM-MS spectrum of WT Aβ 1–40 after exchange (flow 40 mL/min) focusing on 2164–2185 *m/z* range. Vertical axis in the colored panel shows m/z whereas horizontal axis the ion mobility drift time. The colored spots indicate MS peaks with amplitude increasing from purple to yellow. Signals corresponding to MON^2+^, DIM^4+^, TRI^6+^, TET^8+^ were identified based on the analysis of signal spacing in the isotopic envelopes, as shown previously [43]. Cross sections of isotopic envelopes at two drift times indicated by arrows, corresponding to two alternative TRI^6+^ structural forms (insets) show the difference in the distribution of signals after exchange between a more compact form of shorter drift time (upper inset) and a more extended one, characterized by a longer drift time (lower inset). For the extended form the slow exchanging species are minor in contrast to compact form. Projection of this region on the drift time axis (lower panel—vertical axis: signal intensity) shows relative amplitudes of the five signal groups.

The results of partitioning the population of states into several differently exchanging conformational variants are shown in Fig 4A, right panels and in Figure C, panels i-iii in S1 Appendix. Even for monomers, at least five distinct forms are needed to account for the experimentally observed isotopic envelopes after HDX, of which four represent major forms. The four major forms displayed deuterium uptake of 4 D, 10 D, 18 D and 28 D for the 2+ monomer. For lower HDX reagent flow rates (20, 30, 40 mL/min, data not shown), the same five distinct forms were observed but with a reduced labeling compared to the experiment performed at 50 mL/min. For dimers (DIM^4+^), three major forms were populated, with 4 D, 9 D and 14 D exchanged per monomer, while the fast exchanging form with 20 D was minor. For the trimer, forms with 6 D and 10 D predominated among the 5 distinct forms (Fig 4A). In the case of trimers, however, the S/N at flow 50 mL/min was low, decreasing the quality of fit. Additionally, as noted before and confirmed in the present analysis (see also Figure A in S1 Appendix and Fig 2 in Ref. [43]) the TRI^6+^ signal was split into two isotopic envelopes, both corresponding to TRI^6+^, but one of a shorter and the second of a longer drift time in the IM dimension. This result indicated the presence of alternative, more compact and more extended structural forms of a trimer. Interestingly, the deuterium uptake profiles of these two trimeric forms were different (Fig 5). In the case of the compact form, the slower exchanging, better protected species were prominent in the isotopic envelope. These slower exchanging species were minor in the more extended form. Other signals in the IM-MS spectrum, corresponding to different oligomeric forms, were also split, marking the existence of alternative structural states. The analysis of the split signal of DIM^5+^ also showed better protection of the compact form (Figure D, panel i in S1 Appendix). This effect was less pronounced for TET^6+^ (See also Figure D, panel ii in S1 Appendix). More extended oligomeric forms, at least for lower order oligomers, were thus more prone to HDX. Although IM is conducted only a few ms after HDX this may indicate that the differently exchanging forms can evolve into two well defined separate structural variants during the transfer to the point where IM is performed.

The precise intensity distribution of identified forms differed slightly between batch-to-batch replicate analyses (see Figure C, panels i-iii in S1 Appendix), however, the fast exchanging form with approx. 27 D exchanged was reproducibly present in monomers and absent in oligomers. Oligomers are thus characterized by the predomination of forms which exchanged approx. 5, 9 or 14 D with negligible abundance of a fast exchanging form. Our results therefore indicated that in oligomers, a network of side-chain interactions (salt bridges and H-bonds) was organized so that it engaged a substantial fraction of side-chain protons. As compared to 27 D exchanged in the monomeric, fast exchanging form under these conditions, the dominant dimeric forms exchanged 4, 9 or 14 protons, leaving 23, 18 or 13 protons protected, respectively. The majority of non-amide heteroatom-bound side-chain protons in Aβ peptide were located at the N-terminal region (Fig 1), so this network primarily involved side-chains of residues of the N-terminal 16 residues, which contained 19 of the total of 27 exchangeable protons, whereas the C-terminal, longer part contained only 8 such protons.

**Fig 6:**
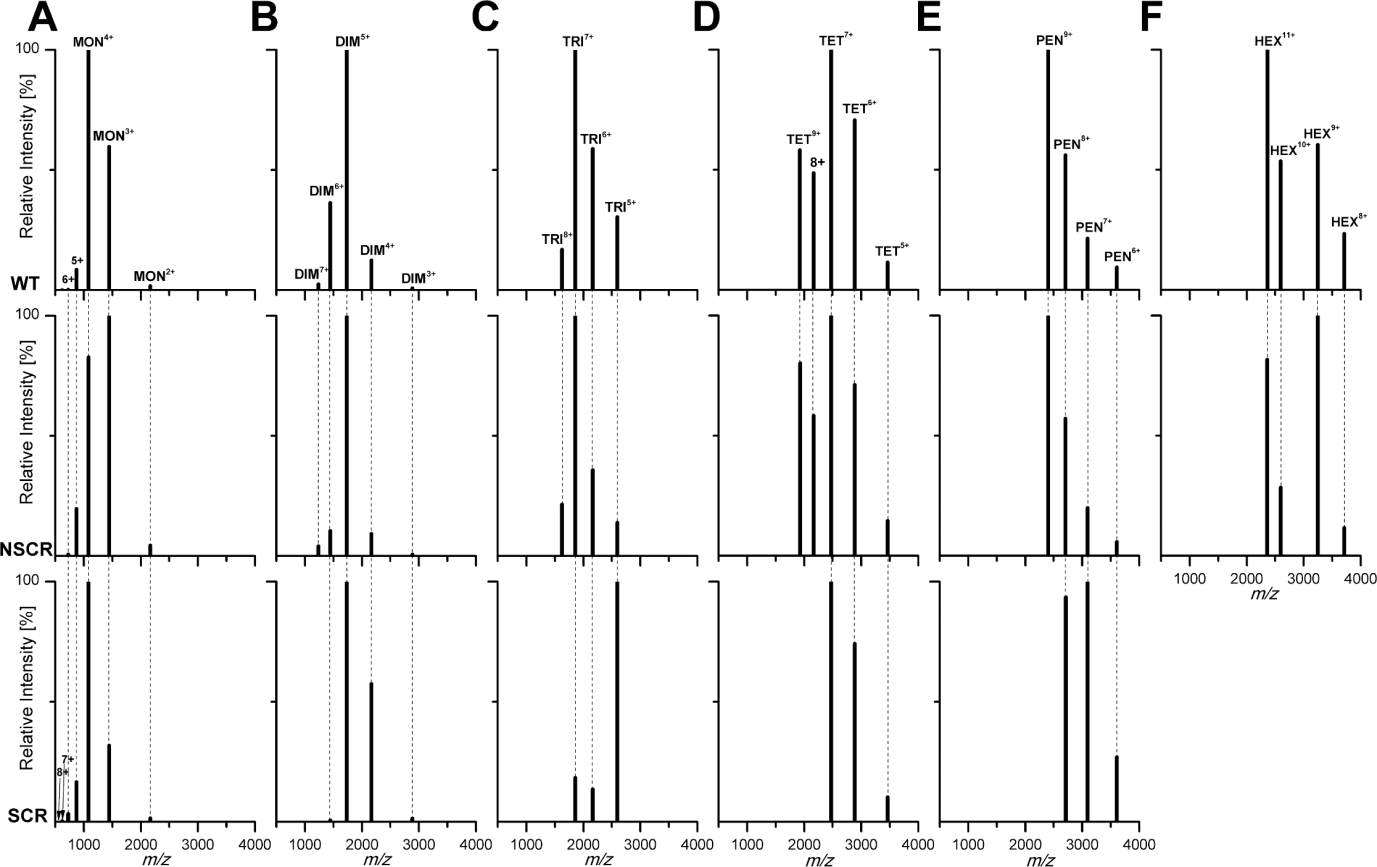
Charge state distributions of MS signals of: (A) monomers, (B) dimers, (C) trimers, (D) tetramers, (E) pentamers, (F) hexamers of WT Aβ 1–40 (upper panels), NSCR Aβ 1–40 (middle panels) and SCR Aβ 1–40 (lower panels), extracted from IM-MS data, with the height of each signal equal to the maximum value of the signal in the corresponding charge envelope. In SCR, DIM^7+^ was missing, DIM^6+^ was less intense, and DIM^4+^ was more intense. The charge state envelope was in some instances (dimers, trimers, pentamers) shifted towards a lower charge in SCR. For trimers, the dominant form was 5+ in SCR and 7+ in both WT and NSCR, while the 8+ form was missing in SCR. TET^8+^ and TET^9+^ were missing in SCR but the remaining 7+, 6+, and 5+ signals retained the same mutual amplitude. A major PEN^9+^ signal in WT and NSCR was missing in SCR, for which PEN^7+^ was predominant. Hexameric signals are absent in SCR.

To check if the network of interactions involving the N-terminal residues depends on peptide sequence or composition we have also studied peptide variants in which the sequence was scrambled. In the first variant, NSCR, only the N-terminal 16 amino acids were scrambled, whereas the sequence at positions 17–40 was not changed, as illustrated in Fig 1A. Interestingly, scrambling of the N-terminus in NSCR did not lead to major changes in the HDX-IM-MS spectra (see below). So, it was necessary to check if this insensitivity to scrambling extends towards the C-terminus. Therefore we have also studied a second variant, SCR, in which the whole Aβ 1–40 sequence was scrambled such that all Aβ amino acids were present but each was placed at a non-native position in the sequence. The SCR sequence was selected based on previously published work [53]. For both scrambled peptides, IM-MS revealed the presence of oligomeric signals. The distribution of oligomeric species observed is best illustrated in plots where the charge state envelopes extracted from IM-MS spectra are shown (Fig 6). Even though the sequences were scrambled, major oligomeric forms still were present, both in NSCR (middle panels) and in SCR (bottom panels). However, IM-MS spectra of SCR and WT Aβ, collected in parallel under the same MS settings, differed in three aspects. First, the higher charged oligomeric states were, in general, missing (or were much less intense) in SCR, compared to WT and NSCR, as described to more detail in the legend of Fig 6. Lower charge forms were present in both scrambled peptides and had the same drift times, and thus the same Ω, as measured for the WT peptide previously [43], while higher charge states were detectable only in WT Aβ oligomers. Interestingly, for monomers the direction of changes was opposite, and for SCR, unique highly charged signals for MON^7+^ and MON^8+^ were detected. Absence of higher charged forms in SCR oligomers may be caused by better shielding of charging protons in SCR oligomers or, alternatively, by their lower stability and therefore easier gas phase dissociation of oligomers into lower oligomeric states. The second difference was that signals corresponding to higher order oligomers, irrelevant of charge, were completely absent in the case of SCR (as shown for hexamers in Fig 6F).

**Fig 7:**
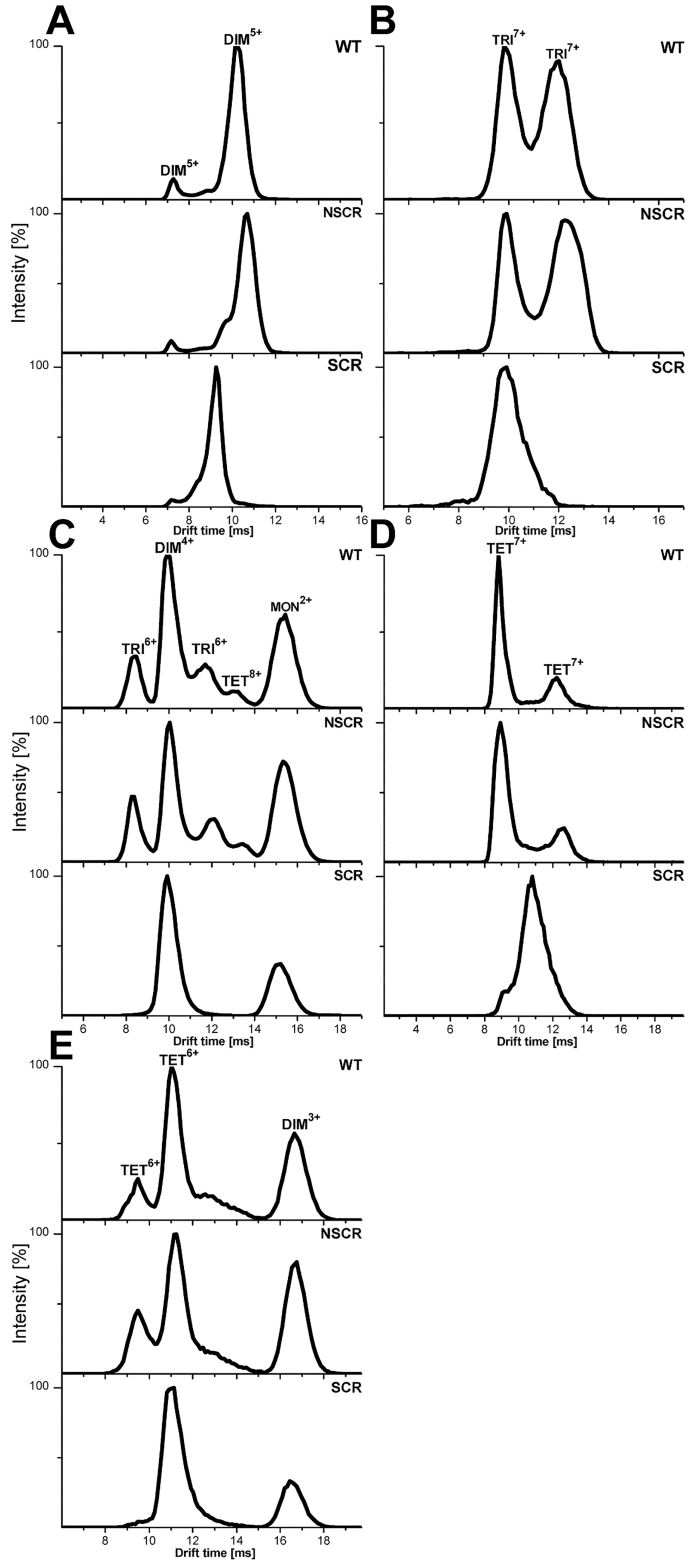
IM-MS drift time profiles of selected signals, corresponding to WT Aβ 1–40 (upper traces), NSCR Aβ 1–40 (middle traces) and SCR Aβ 1–40 (lower traces) oligomers. For each selected *m/z* region, a profile of molecular species present at different drift times in the IM T-wave is shown, with each species assigned to a particular oligomeric charge state. Some profiles contain a single peak, indicating the presence of one form of a single collisional cross section, whereas other signals are split in the domain of the drift time into multiple species. (A) DIM^5+^—the dominant, extended form becomes more similar to the compact form in SCR. For TET^7+^ (D) and TET^6+^ (E), alternative structural forms of WT Aβ collapse in SCR to a single major form with intermediate drift time (and thus intermediate Ω), while TRI^7+^ in SCR (B) exhibits a single form with the drift time closer to the compact form in WT. The split TRI^6+^ signals as well as the TET^8+^ signal (C), which are clear in WT and NSCR, become very weak in SCR. Signals were assigned to species as described before in Ref [43].

The third difference between the IM-MS spectra of SCR and WT Aβ 1–40 was that the splits in drift times of the signals from oligomers of the same number of monomers, i.e., the same order, caused by the presence of alternative conformational states, that have been commonly observed with increasing charge in WT Aβ 1–40 [43], were not observed for SCR (Fig 7). Alternative structural forms of WT Aβ 1–40 collapse in SCR either to a single major form with intermediate drift time (and thus intermediate Ω), as in TET^7+^ (7D), TET^6+^ (7E), or a form with the drift time closer to the compact form in WT, as for TRI^7+^ (7B), or the split states disappear completely as for TRI^6+^ (7C). In DIM^5+^, the dominating form became more like the compact form (Fig 7A). To facilitate the comparison with the work of others [40,44], we also observed the same effect using negative ionization (see 2D map of 2165 region in Figure E in S1 Appendix). The ability to form alternative, slowly interconverting states in WT, which were absent in SCR, seems thus to be encoded in the partitioning of the sequence into a hydrophobic C-terminal and hydrophilic N-terminal region. Nevertheless, the N-terminal region could be scrambled, as in NSCR, and the alternative slow interconverting states were still retained. These potentially important faculties were lost upon complete scrambling in SCR, indicating that the partition into two regions affects the structural properties of oligomers.

### DLS and CD experiments

**Fig 8:**
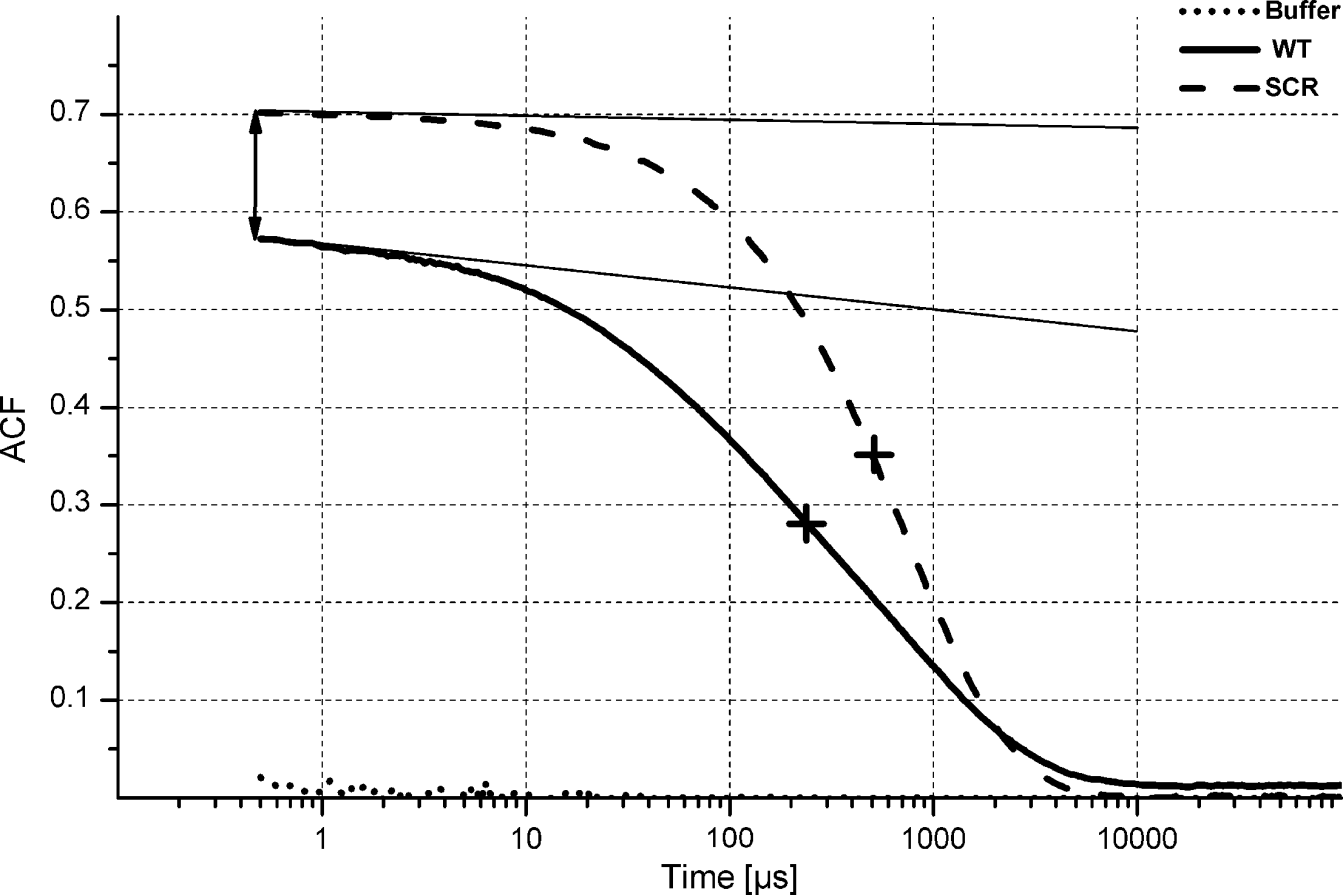
DLS experiment of WT Aβ 1–40 and SCR Aβ 1–40. Representative autocorrelation function (ACF), directly adapted from dynamic light scattering data obtained for WT Aβ 1–40 (line) and SCR Aβ 1–40 (dashes). The autocorrelation function decays in the range of 1 down to 0, and the gap remaining to 1 observed at the shortest correlation time is indicative for the existence of monomeric or low-order oligomers, which remain undetectable. The short-correlation-time asymptote of ACF is marked by thin solid lines. The mid-point of an ACF decay is marked by the cross.

To check if the differences between SCR and WT oligomeric distributions could also be directly observed in solution we carried out DLS and CD experiments (Fig 8 and Figures F and G in S1 Appendix). The analysis of DLS data (Fig 8 and Figure G in S1 Appendix), namely the Autocorrelation Function (ACF) of the scattered light, also revealed significant differences. The short-correlation-time asymptote of ACF for SCR is nearly horizontal, which is not the case for WT (marked by thin, solid lines), indicating a smaller fraction of low order oligomeric forms in SCR. It should be however noted that the autocorrelation time of 10 *μ*s roughly corresponds to objects of the mass ∼10 kDa (i.e. Aβ dimmers/tetramers), while autocorrelation time of 30 *μ*s is indicative for ∼100 kDa oligomers (∼20-mers). Consequently, oligomers revealed at larger autocorrelation times in DLS are undetectable in MS experiment. In agreement, signal value at the shortest observable correlation time (at the intersection with vertical axis, indicated with the arrows) is higher for SCR, also indicating smaller fraction of monomers and very small oligomers. For SCR ACF revealed a more narrow transition in the correlation time domain, indicating a more homogeneous distribution than for WT, with a smaller amount of low order and extremely high order oligomers. The shift in a mid-point of an ACF decay, located at ∼293 *μ*s for WT and ∼409 *μ*s for SCR (marked by the crosses), indicated the differences in the mean size of oligomers, which remained, on average, larger for the SCR variant. CD spectrum of SCR, although mainly a random coil, revealed a stronger 222 nm band than WT, indicating also structural differences between WT and SCR (Figure F in S1 Appendix). In general, DLS indicated more narrow distribution of high-order oligomeric forms in SCR with both low-order and high-order forms depleted relative to WT. This correlates well with absence of larger oligomeric forms in MS spectra of SCR.

The increased protection of protons in oligomers was much less pronounced in SCR (Fig 3C) and more pronounced in NSCR (Fig 3B) than in WT (Fig 3A). Scrambling the entire sequence and spreading the hydrophilic residues along the whole sequence led to similar deuterium uptake in monomers and oligomers. Interestingly, for SCR monomers, a much smaller uptake was observed (Fig 4B, Figure C, panels iv-vi in S1 Appendix), as compared to WT (Fig 4A, Figure C, panels i-iii in S1 Appendix) with a clear lack of the fast exchanging form (uptake of 17–28 D). In SCR, slow exchanging forms (3–10 D) are the major forms for monomers, dimers (middle panels), and trimers (lower panels), with faster exchanging forms (11–16 D) being in the minority. Possibly, spreading the hydrophilic residues along the entire sequence provides a better opportunity to configure them in a way that results in more efficient shielding from deuterium exchange (i.e., solvation in the gas phase of exchangeable charged sites by internal hydrogen-bonding and electrostatic interactions) [39].

## Discussion

**Fig 9:**
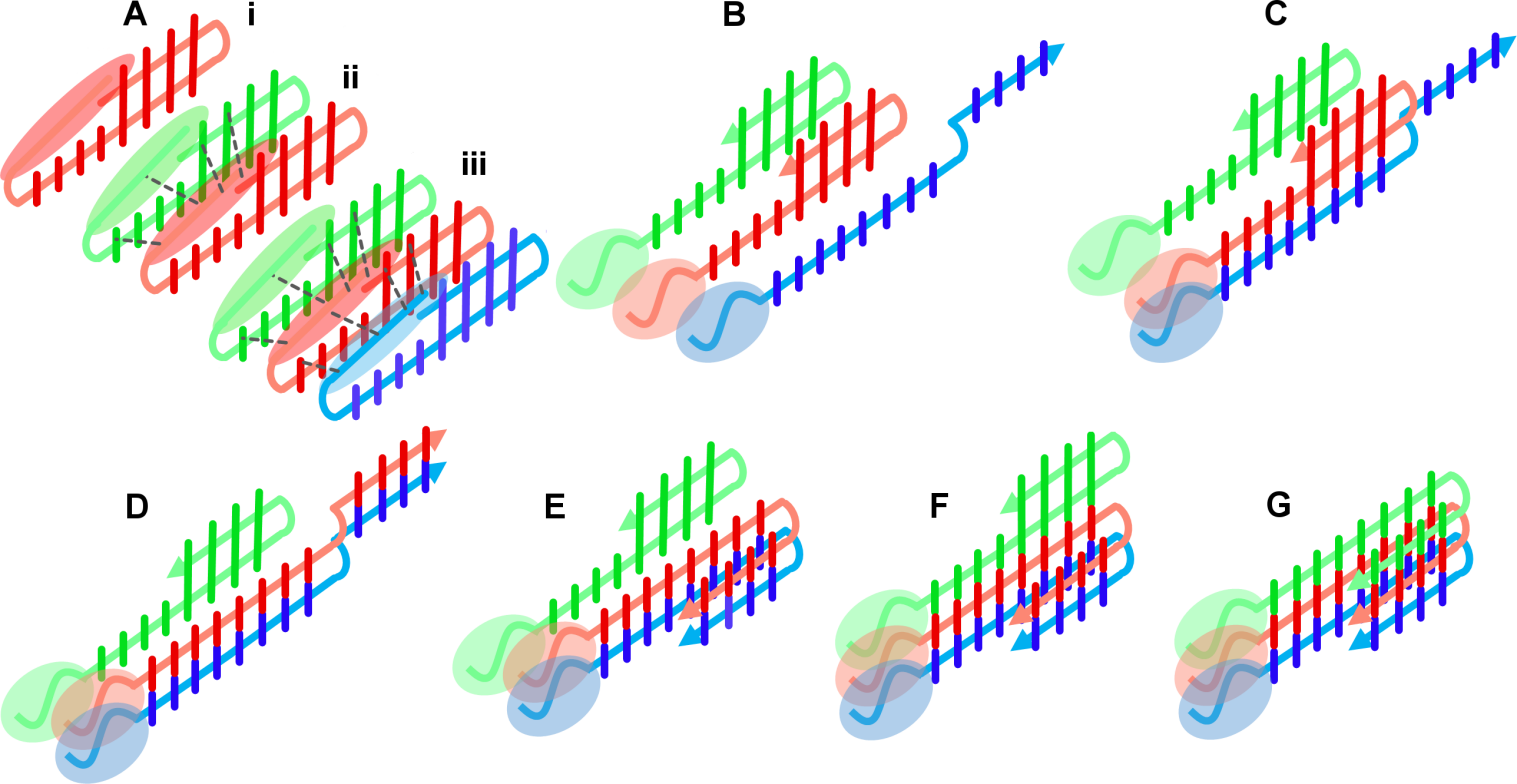
(A) Schematic representation of a build-up of a potential network of intermolecular interactions in oligomers upon transition from monomers (Ai) to dimers (Aii) and trimers (Aiii). Since N-terminus does not necessarily maintain a single conformation, most probably interacting in a non-specific way mediated by multiple chain arrangements, it cannot be represented as a single conformation. Dashed, grey lines mark new interactions, absent in monomers. Simplified scheme illustrating the main steps in a hypothetical scenario of a conversion of a stacked β-hairpin oligomer, shown in (Aiii), consisting of three units into a parallel β-structure (G). In the first step a single edge monomer (blue) opens into and extended state (B) and releases its intramolecular H-bond donors and acceptors for the intermolecular interactions with the free H-bonding partners of the neighboring hairpin (red, panel C). This provides the opportunity for propagation of intermolecular parallel H-bonds along the entire sequence between the first two monomers (D). A compact state can be regained (E) leading to dimeric nucleus of a parallel β-structure. This exposes the unsaturated potential H-bonding sites of the second hairpin (red) for parallel H-bonding with the next neighboring hairpin (green, panel F) and leading to incorporation of the third unit of the intermolecular parallel β-structure (G). Scheme was tested possible in molecular dynamics simulations (Figure H in S1 Appendix, S1 Movie).

Our work indicated that the N-terminal part of Aβ peptide is not a neutral bystander, but it rather participates in the interaction networks shaping the oligomeric equilibria. Side-chain residues of the region 1–16 are entangled in interactions which are more frequent in oligomers than in monomers (see scheme in Fig 9A). The analysis of scrambled sequences showed that maintaining the bipartite character of the Aβ peptide sequence, consisting of a hydrophobic C-terminal region and a hydrophilic N-terminal region, was necessary for the ability of the peptide to form higher order oligomers with alternative structures, compact and open. Thus, a new insight into the role of the N-terminus of the Aβ peptide in oligomerization has been obtained. Previous classic HDX study [54] led to the conclusion that the N-terminal part (res. 1–16) retained nearly full solvent accessibility until the stage of fibrils. However, when a shorter labeling time (50 ms) was used [55], a significant protection of N-terminal amides (res. 1–11) became apparent (see Fig 8 in Ref. [55]). A fibril with several ordered N-terminal residues has previously been reported [12]. In the same study, HDX monitored by NMR revealed exchange at a set of N-terminal amides, contrasting to strong stability of the C-terminal part and indicating higher dynamics of the N-terminus than the core of the amyloid. Molecular dynamic studies that followed [56] revealed the involvement of the N-terminus in inter-fibril interactions and interactions of R5-S8 region with E22-N27 region. Also, in a recent fibril structure the N-terminus is structured [57], however these fibrils were obtained by incubation at low pH in the presence of high concentration of organic co-solvent. With known sensitivity of Aβ peptide structures to external conditions the relevance of this structure to native is difficult to assess. Site-specific backbone dynamics was directly measured by solid state NMR in mature fibrils of Aβ 1–40 [58] and protofibrils [59]. Order parameters were found to be only slightly lower in the N-terminal part than in the core region of the amyloid, implicating substantially restricted motions also in the N-terminal region, with Asp1, Ala2 revealing higher order parameters than Glu3, both for protofibrils and fibrils. In case of Asp1 and Phe4 the order parameter is higher in protofibrils than fibrils, suggesting a role of the N-terminus in fibril maturation. Studying oligomeric fractions of Aβ 1–40, stabilized by addition of alcoholic co-solvent, Fändrich group used magic angle spinning solid-state NMR to reveal the involvement of the N-terminal residues 4, 7–12 in a β-strand structure [60]. In agreement, the involvement of the N-terminal residues in oligomer-stabilizing interactions has been found in the presented work.

### Biological evidence for the importance of the N-terminal region

Mutations in the N-terminal region of Aβ peptide may lead to familial AD (e.g., A2V, H6R-English, D7N-Tottori, D7H-Taiwanese) or may be protective (A2T) [61]. Also, the N-terminal sequence contains amino acids responsible for metal ion binding, a crucial factor shaping the Aβ peptide aggregation properties [62,63]. Pike et al. observed *in vitro* that deletion of residues 1–16 enhanced aggregation of Aβ peptide variants [64]. On the other hand, such deletion is the result of an endogenous, “non-amyloidogenic” amyloid precursor protein processing pathway, leading to formation of the “p3” peptide [64]. p3 was found in pre-amyloid deposits in Down's syndrome brains [65–67], but also in plaques and diffuse deposits in selected areas of AD brain [27]. Although being a product of the “non-amyloidogenic” pathway, it is not necessarily non-amyloidogenic [64,66,68]. p3 can adopt a β-sheet conformation [64,66], indicating that this peptide is able to form some amyloid-like fibrils, although it preferentially aggregates into amorphous forms. Current knowledge on biological properties of p3 is limited. Some proapoptotic [69] and proinflammatory [70] properties have been indicated. Dickson et al. showed that the N-terminal region is responsible for activation and recruitment of glia to senile plaques [71]. Also, an internal 25–35 fragment of p3 was shown to exert neurotoxicity [72]. On the other hand, Walsh et al. revealed that p3 solutions do not affect synaptic function [28]. To some extent, p3 seems to retain both fibrillogenic and neurotoxic properties, suggesting that the presence of N-terminal residues enhances these properties. The involvement of N-terminal residues in oligomer maturation pathways, shown here, may underlie known differences in aggregational and biological properties of Aβ peptide.

### Gas phase evolution of solution structures

Oligomeric species of peptides under study were observed both in the gas-phase by HDX-IM-MS but also in solution, using DLS. For SCR, DLS showed a decreased fraction of low order species in agreement with the lack of oligomeric signals form (pentamers and larger oligomers) observed in the gas phase. Following dehydration, for tens to several hundreds of milliseconds after ionization, structures evolve towards a gas phase stable state [38–40]. Molecular simulations suggest that at the timescale of picoseconds side-chains rearrange into a network of electrostatic salt bridges, much strengthened in the absence of water [73]. These interactions may “cross-link” and temporarily stabilize the native-like backbone fold, which can remain unchanged for up to tens of milliseconds. On the timescale of seconds backbone rearrangements enable further collapse into compact gas-stable structures. Thus, a basic requirement for the use of gas-phase HDX-MS to reflect on solution-phase protein conformers is to complete the exchange reaction within a few tens of milliseconds after ionization [37,51]. In the chosen gas-phase HDX-MS setup, deuterium labeling was obtained in the submillisecond timescale during the transfer of ions between the cone and the source T-Wave ion guide. Gas-phase HDX-MS executed at such short timescales may thus report on solution-phase structures and structural differences between Aβ species [36,37,45,51], however the direct correspondence of structures detected in the gas phase and in solution cannot be claimed.

### Ionic interactions–shift in charge density

Interaction networks stabilizing protein structure may entangle the potential protonation sites, so native protein structures, tested in ESI-MS, carry less charge than their denatured counterparts. We have observed in this work (Fig 6) and in previous investigations (see S1 Table in Ref. [43]) that charge per monomer (CpM) decreases with the order of oligomer. For the dimer, the signals correspond to CpM of 1.5–2.5, while for hexamers only signals corresponding to CpM 1.8 (HEX^11+^) down to CpM 1.17 (HEX^7+^) were observed, whereas signals of HEX^12+^ and higher charges were not observed. The absence of HEX^+12^ signal in the isobaric MON^2+^, DIM^4+^, TRI^6+^, TET^8+^ group of signals was previously interpreted as indicating lack of hexamers in the oligomer population [44], however it is rather a consequence of lower charging of hexamers with clear hexameric signals detectable at lower charge (+7, +8, +9) values [43]. In agreement with these earlier observations, our present work confirmed the decreased availability for exchange of side-chain protons in oligomers as compared to monomers.

### Model of antiparallel-parallel transition

Our work showed that the ability to form alternative conformational states (compact and extended), also observed previously [43], was lost upon scrambling of the entire amino acid sequence, but not by scrambling only the N-terminus amino acids. This indicates that partitioning of the Aβ sequence into two regions, a hydrophilic N-terminal part and the hydrophobic remainder, may be necessary to enable Aβ peptide to form alternative conformational states. These states were previously assigned to an open state and a closed β-sheet sandwich, leading to an oligomer model (see Fig 4 in Ref. [43]), confirmed by mutagenesis at the turn region of residues 23–28 [50].

The existence of such compact-extended equilibrium may also provide a mechanistic explanation for the transition between an antiparallel stacked-β-hairpin oligomer, suggested previously as an initial oligomeric state (see Fig 4 in Ref. [25]), into a parallel β-sheet oligomer, constituting fibrils. This concept was supported by experimental data for Aβ 1–42 [19,74]. Also, disulfide bonded, cross-linked Aβ peptide variants, designed to stabilize a β-hairpin, were shown to spontaneously form stable oligomers and protofibrils, but were unable to convert into amyloid fibrils [75,76]. Antiparallel β-structures have been observed for other amyloid-forming proteins, so reorientation of β-strand directionality could be a generic mechanism of cross β-sheet formation.

Parallel-antiparallel conversion requires rotation of chains by 90° along the axis defined by the peptide backbone, so that anti-parallel intramolecular H-bonding of stacked hairpins becomes parallel and intermolecular. Such conversion would not be possible without the release of intramolecular H-bonds stabilizing the hairpin structure. The presence of alternative, extended forms of an oligomer provides a possible clue for a hypothetical scenario, sketched below, in which chains are able to switch their H-bonding partners within an oligomer without disassembly into monomers. To investigate this possibility at the molecular level, we have designed a hypothetical model of the transition presented in Fig 9B in the simplified form and performed molecular modeling and dynamics simulations (for more detailed information see Materials and Methods, Text A in S1 Appendix) simulating this transition. Obtained MD trajectory (Figure H in S1 Appendix and S1 Movie) verifies the possibility of the conversion of a stacked β-hairpin oligomer, consisting of three units (Fig 9Aiii), into a parallel β-structure (Fig 9G). Corresponding MD snapshots are shown in Figure H in S1 Appendix. The conversion can be nucleated if a single edge monomer (marked blue in Fig 9, Figure H in S1 Appendix) opens into and extended state (Fig 9B, Figure H, panel ii in S1 Appendix), releasing its intramolecular H-bond donors and acceptors for the intermolecular interactions with the free H-bonding partners of the neighboring hairpin (marked red in Fig 9C, Figure H, panel ii in S1 Appendix). This provides the opportunity for propagation of intermolecular parallel H-bonds along the entire sequence between the first two monomers (Fig 9D, Figure H, panel iii in S1 Appendix). A dimeric nucleus of a parallel β-structure can then be formed (Fig 9E, Figure H, panel iv in S1 Appendix), exposing the unsaturated potential H-bonding sites of the second hairpin (red) for parallel H-bonding with the next neighboring hairpin (green in Fig 9F) and leading to incorporation of the third unit of the intermolecular parallel β-structure (Fig 9G). The conversion can thus be propagated across the oligomer, as illustrated by the snapshots (Figure H, panels v-vii in S1 Appendix) from the molecular dynamics trajectory (S1 Movie). Antiparallel β-sheet structure has also been observed for non-Aβ oligomers [77,78], therefore a conversion step into cross-β structure and the proposed molecular mechanism may be of a more general nature.

All these steps can, in principle, happen between monomeric hairpins. However, the preformed stacked β-hairpin oligomer provides the entropic trap that makes this transition more probable, and thus faster. Critically, the entropic trap could be supported by a network of relatively non-specific interactions with the N-terminal residues (marked by color ellipses in Fig 9 and omitted in Figure H in S1 Appendix, S1 Movie for clarity), which do not directly participate in the transition but protect the oligomer from loss of monomers in the transition states. It can thus be hypothesized that the N-terminal region side-chain interactions identified in this study might provide necessary support during this transition, protecting the intermediate open state of an oligomer from decomposition into monomers and explaining why p3 peptide is less efficient in fibrillization. As a consequence, the uniqueness of the Aβ sequence would lie in its tripartite composition marked by the N-terminal hydrophilic part and two hydrophobic stretches separated by a turn. The N-terminal region may play a role as a sort of “internal chaperone” providing a transitional network of interactions which allows the integrity of the oligomer to be retained during the transformation. Such a mechanism, provided by intrinsically disordered regions, has been observed, for instance, in an intermediate filament maturation pathway [79].

## Conclusions

N-Terminal hydrophilic amino acids of the Aβ peptide seem to play an important role in shaping its neurotoxicity, since the “non-amyloidogenic” processing variant of Aβ, p3 peptide, truncated N-terminally by 16 amino acids, is believed not to be neurotoxic. The presented work provides the first experimental evidence for the involvement of the hydrophilic residues from the N-terminal part into a network of interactions stabilizing oligomeric structures. We have also developed a molecular model which provides a possible clue for the role of these interactions in the maturation pathway of the fibrils.

## Supporting information

S1 Appendix Table A summarizing recent studies of Aβ oligomers, Figures A-H and the MD simulations description.
(DOCX)

S1 Movie Snapshots of the molecular dynamics trajectory visualizing a hypothetical pathway of the incorporation a stacked β-hairpin into parallel β-structure oligomer. Web Enhanced. A WEO is available in the HTML version of the paper.
(MP4)

## Abbreviations

Aβ: amyloid β peptide
AD: Alzheimer's disease
IM: ion mobility separation
SCR: scrambled
NSCR: N-terminally scrambled
ESI: electrospray ionization
HDX-MS: hydrogen deuterium exchange mass spectrometry
ETD: electron transfer dissociation
DLS: dynamic light scattering
MD: molecular dynamics
FWHM: full width at half maximum
CpM: charge per monomer
LMW: low-molecular-weight
SD: standard deviation
SOS: sum of squares
SLS: static light scattering.
