# Supplementary material for: Side-chain moieties from the N-terminal region of Aβ are Involved in an oligomer-stabilizing network of interactions
Source: PLoS One. 2018 Aug 6;13(8):e0201761. doi: 10.1371/journal.pone.0201761 (PMC6078298; doi:10.1371/journal.pone.0201761)
Supplement: S1 Appendix — Table A summarizing recent studies of Aβ oligomers, Figures A-H and the MD simulations description. (DOCX) [file pone.0201761.s001.docx]

**S1 Appendix**

**Table A**

| **Stabilization method/modification** | **Oligomer type obtained** | **Peptide variant** | **Structural highlight** | **Structural method** | **Publication** |
| --- | --- | --- | --- | --- | --- |
| Dilute SDS incubation | 60 kDa globulomer | 1-42 |  |  | [1] |
| Low temperature incubation | Spherical oligomers | 1-40 | In-register parallel β-sheet | ssNMR, TEM | [2] |
| Phage display selected Affibody | Monomer | 1-40 | β-hairpin | NMR | [3] |
| Low temperature incubation | Oligomers | 1-42 | Antiparallel β-sheet | ATR FTIR, AFM, HDX | [4] |
| PICUP | Low-order oligomers | 1-40 |  | EM, AFM, CD | [5] |
| SDS incubation | Preglobulomer and globulomer | N-Met 1-42 | Mixed parallel and antiparallel β-sheet structure | NMR, AFM | [6] |
| Low temperature incubation | Low-order oligomers | 1-40 | β-sheet structure in C-terminal region | HDX-MS, CD | [7] |
| Disulfide X-link A2C, C-term | Protofibril-like aggregate | 1-40 |  | CD, TEM | [8] |
| Low temperature, low salt incubation | Disc-shaped pentamer | 1-42 | Loosely aggregated strands | ssNMR, TEM, FTIR, AFM | [9] |
| Intramolecular disulfide | Various oligomeric fractions | 1-40 and 1-42 | β-sheet | CD, TEM | [10] |
| Low temperature incubation | Spherical oligomers | 1-42 | Protection of all amides but the turn residues 25-28 | HDX-MS | [11] |
| N-terminal Cys-Cys motif | Spherical oligomers | 1-40 |  | AFM, ThT fluorescence, | [12] |
| BA10 antibody | protofibrils | 1-40 | Intramolecular β-strands | ssNMR | [13] |
| Dilute ammonium hydroxide | Protofilaments | 1-42 | Antiparallel β-strands wrapped into a superhelix | Powder X-ray, EM, CD, FTIR | [14] |
| SDS incubation | Preglobulomer, globulomer | 1-42 | Antiparallel β-sheet | SDSLEPR | [15] |
| Dilute SDS incubation | 150 kDa oligomer | 1-42 | Antiparallel β-sheet | ssNMR | [16,17] |
| Disulfide X-link A21C | Protofibril | 1-42 | Hexameric barrel | ssNMR, modelling | [18] |
| Low temperature incubation | Amylospheroid | 1-42 | Parallel β-sheet | ssNMR, TEM | [19] |
| PICUP | Low-order oligomers | 1-42 | N-terminus involved in oligomerization | AFM, DLS, SPR | [20] |
| CDR3 loop of IgNAR | Dimer and tetramer | 18–41 | Noncross β-sheet | X-ray, DLS | [21] |
| Im7, WO2 antibody Fab fragment | Monomer | 10–16 | β-strand, turn/PPII-helix | X-ray | [22] |

**Table A.** The summary of major attempts to verify the structure of Aβ of oligomers and protofibrils using different methodologies.

**Figure A**


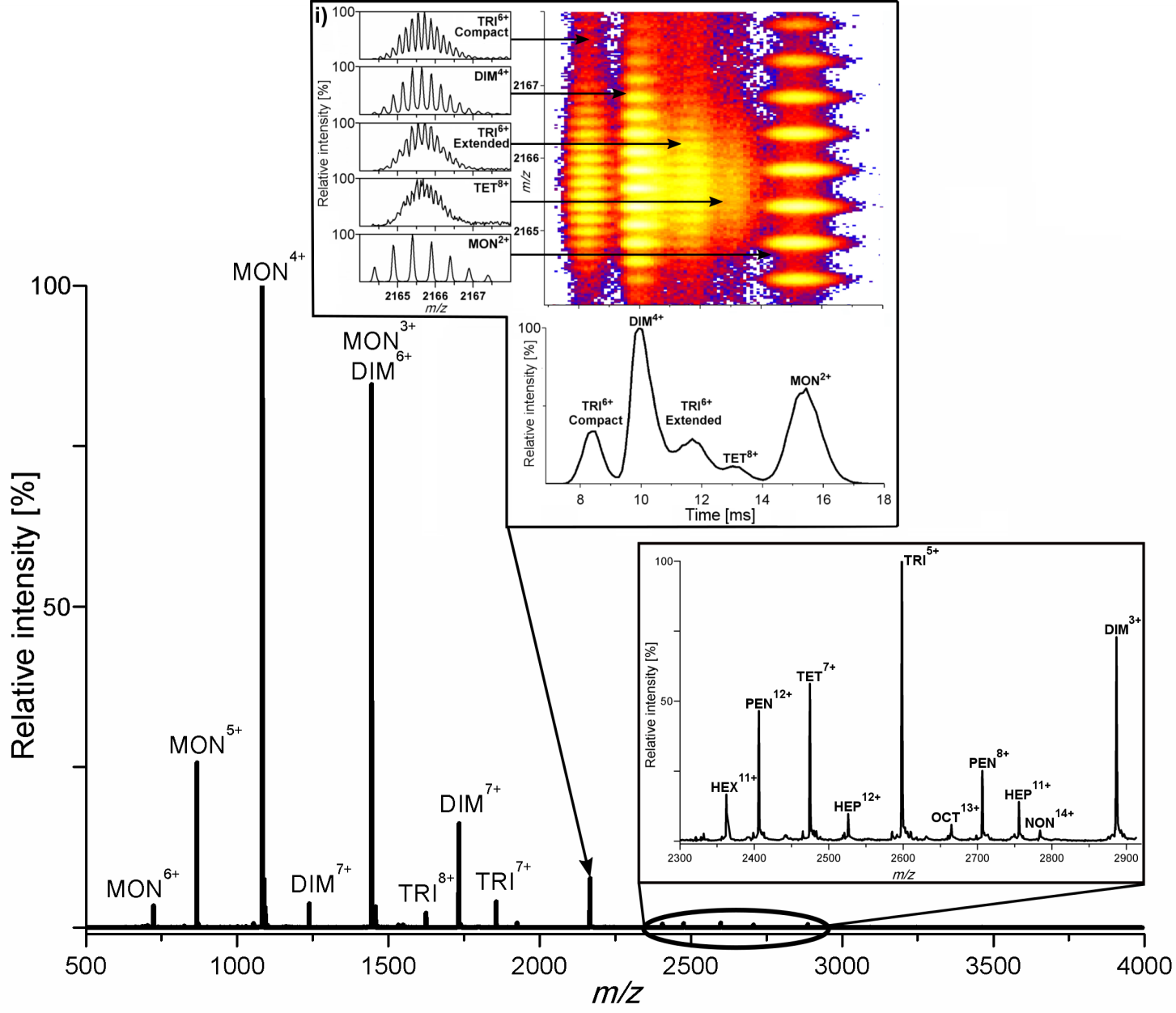


**Figure A.** Native ESI-MS spectrum of WT Aβ 1-40 repeated as a control for the present project using the protocol described previously [23]. In the spectrum, signals expected for monomeric species are accompanied by numerous signals corresponding to oligomeric species (each oligomeric signal with identity tag). Enlarged region 2300-2900 is shown in inset. Panel (i) shows the enlarged fragment of a two dimensional IM-MS spectrum of WT Aβ 1-40, focusing on 2164-2185 m/z range. In this region of the native MS spectrum signals of multiple isobaric species overlay into a single isotopic envelope. In the ion mobility dimension (horizontal axis) separate signals of these species are resolved (lower panel) corresponding to MON^2+^, DIM^4+^, TRI^6+^compact, TRI^6+^extended, TET^8+^, as marked. The signal amplitude is color-coded, increasing from purple (low intensity) to bright yellow (high intensity). Cross sections in the m/z domain (vertical axis) of isotopic envelopes at different drift times are indicated by arrows.

**Figure B**


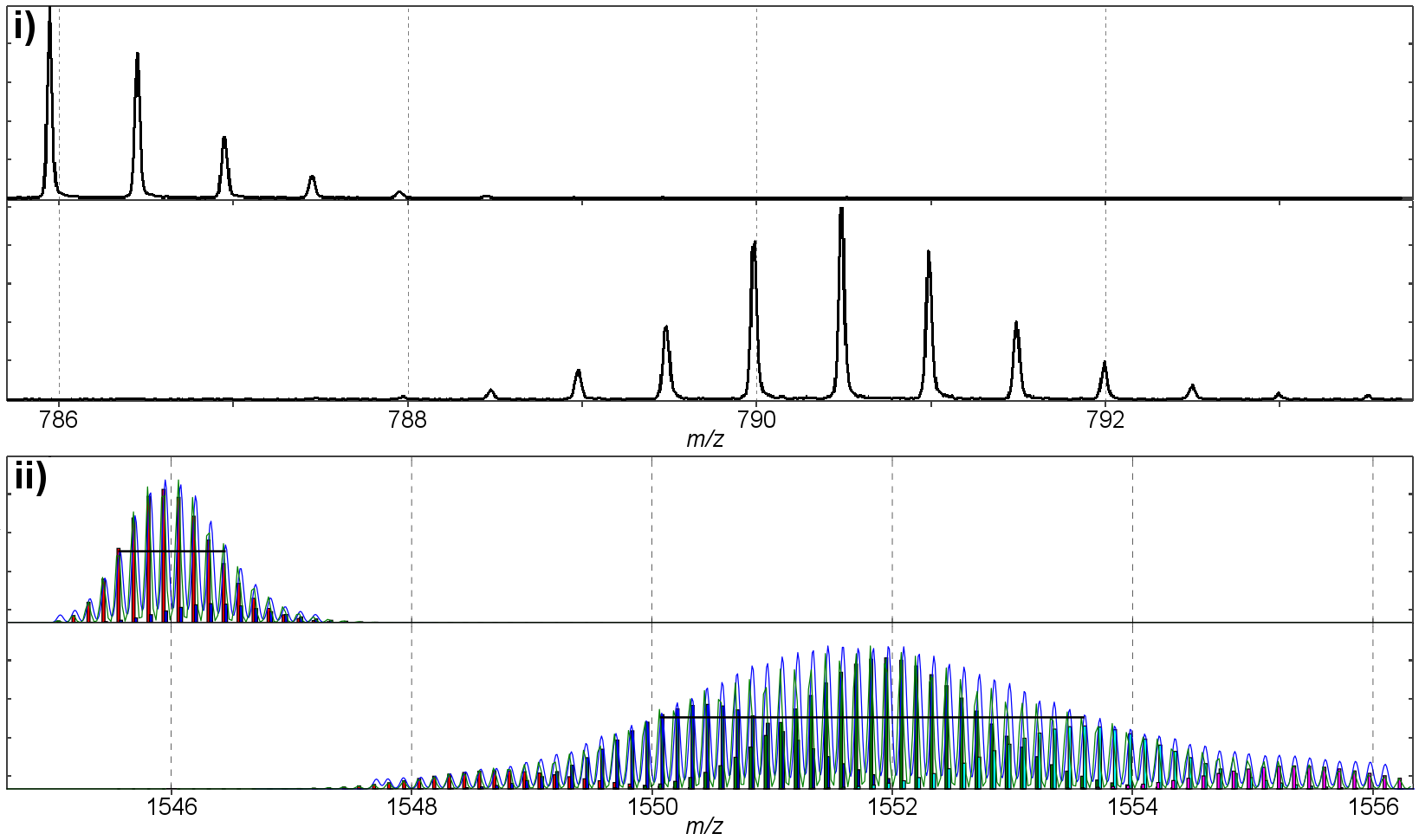


**Figure B.** Isotopic envelopes of the Glu-fibrinopeptide B (charge 2+) (i) and the protein Cytochrome c (charge state 8+) (ii) as a function of gas-flow (0-50 mL/min) across ND_3_/D_2_O reagent. Glu-fibrinopeptide B after HDX presents no widening of the isotopic envelope. For Cytochrome c the full width at half maximum (FWHM) for an isotopic envelope distribution is marked with a solid line (3.75 m/z) and decomposition into a family of uniform conformational states is also shown by colored bars.

**Figure C**


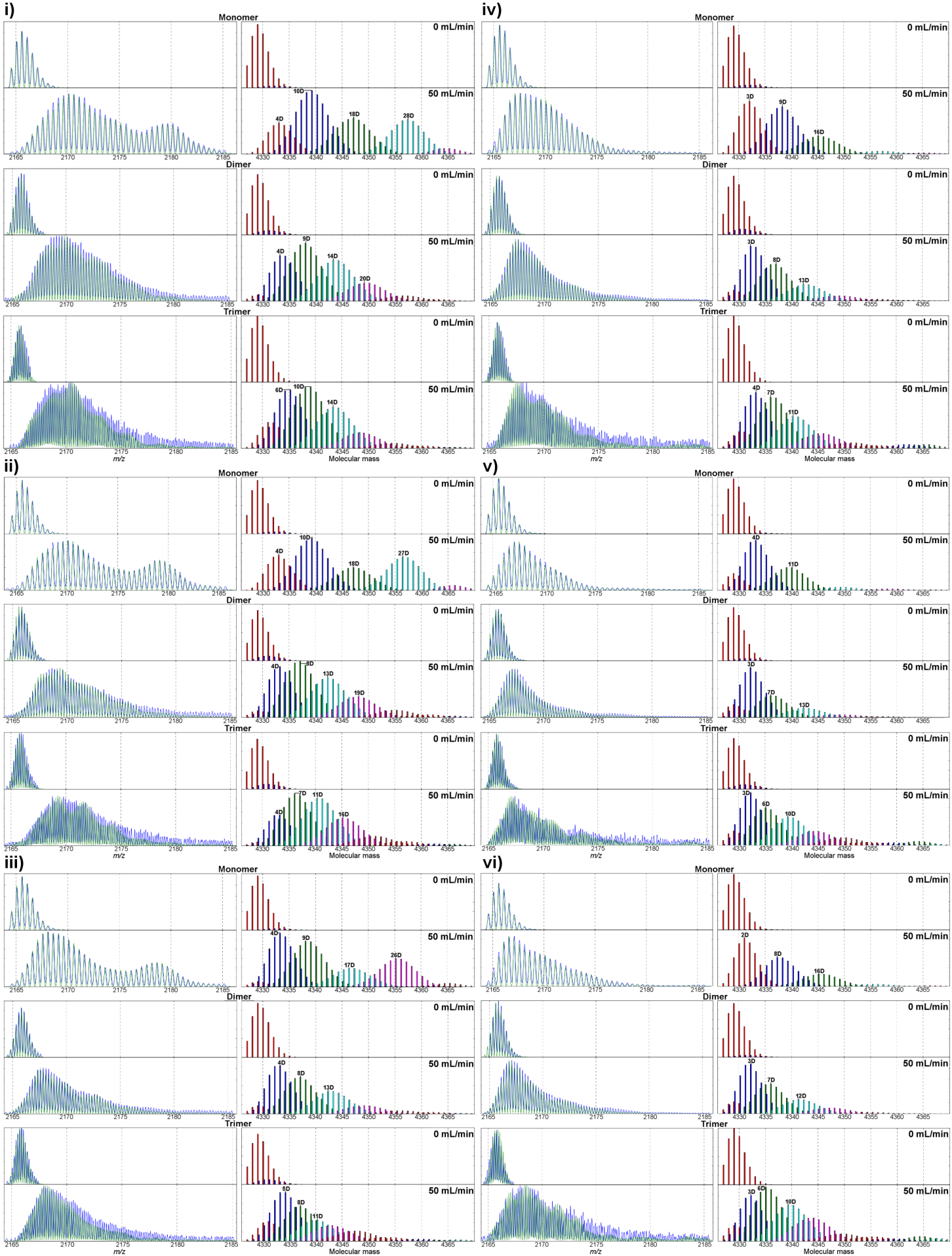


**Figure C.** Analysis of the isotopic envelopes after gas-phase HDX-MS for selected signals of species bearing the same charge per monomer, namely MON^2+^, DIM^4+^ and TRI^6+^ (2164-2185 m/z range) comparing three replicates WT Aβ 1-40 (i-iii) and SCR Aβ 1-40 (iv-vi). Left panels - isotopic envelopes corresponding to MON^2+^ (upper panels), DIM^4+^ (middle panels), TRI^6+^ (lower panels), for make-up gas flow of 50 mL/min in the presence of ND_3_/D_2_O reagent. Right panels - decomposition of the experimental isotopic envelope into a family of isotopic envelopes of FWHM expected for a single conformational state. The spectra were recalculated from *m/z* domain, (shown in left panels) to the domain of molecular mass of a monomeric unit in an oligomer (shown in right panels).

**Figure D**


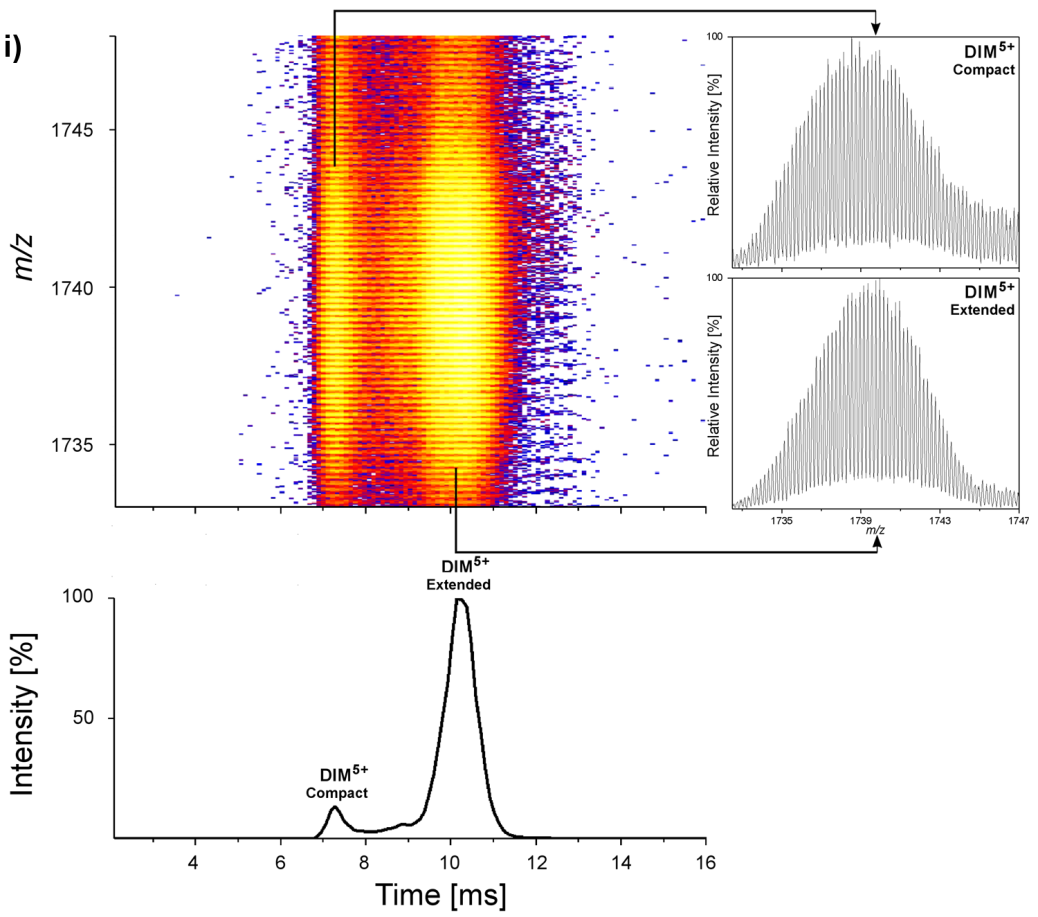


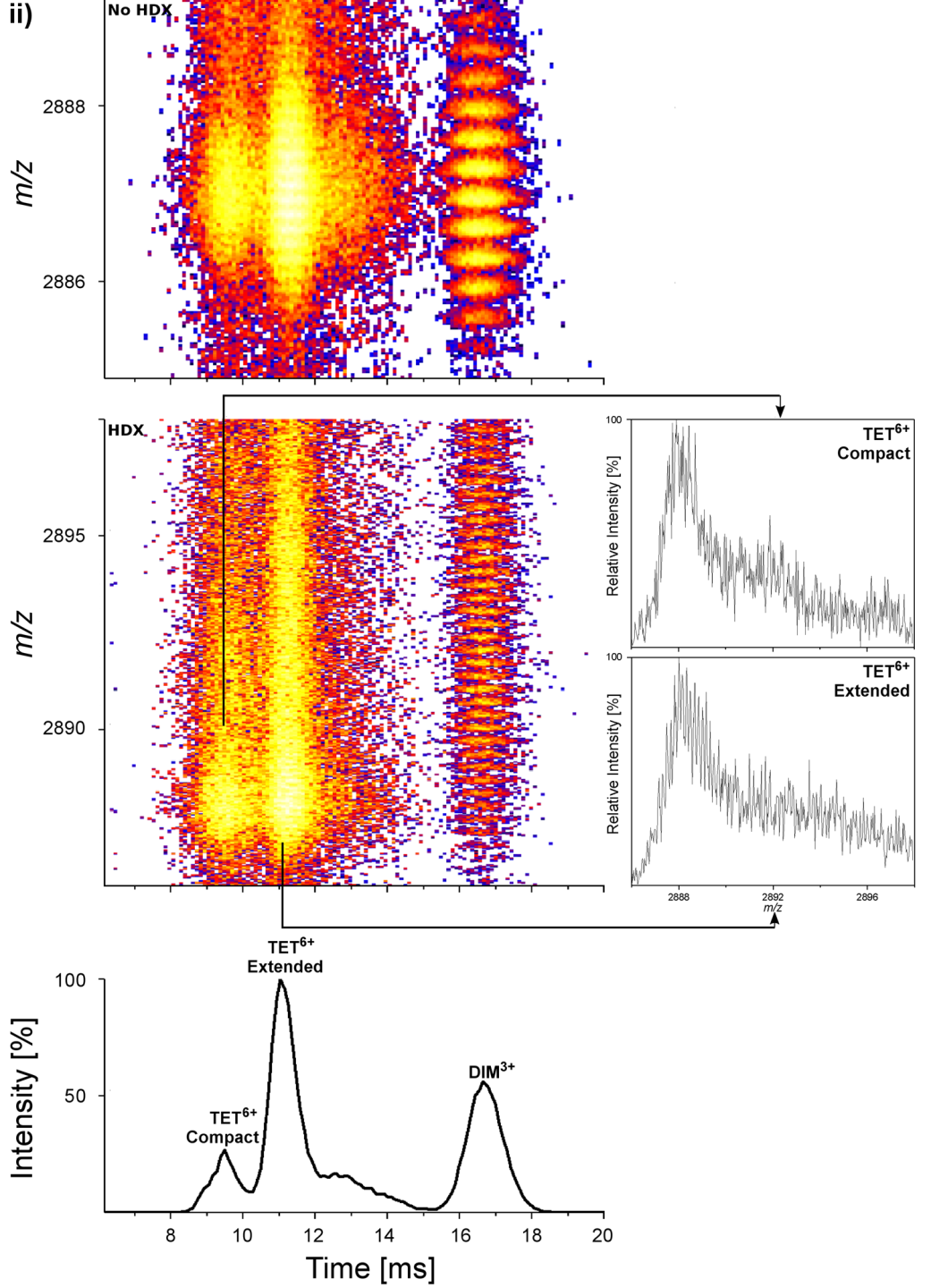


**Figure D.** Enlarged fragment of IM-MS spectrum of WT Aβ 1-40 after exchange (flow 40 mL/min) focusing on DIM^5+^ 1732-1746 *m/z* range (i) and TET^6+^ 2885-2900 *m/z* range (ii). The colored spots indicate MS peaks with amplitude increasing from purple to yellow. Cross sections of isotopic envelopes after HDX at two drift times indicated by arrows, corresponding to two alternative DIM^5+^ and TET^6+^ structural forms show the distribution of signals after exchange for a more compact form of shorter drift time (upper insets) and a more extended one, characterized by a longer drift time (lower insets). Projection of these regions on the drift time axis (vertical axis: signal intensity) shows relative amplitudes of the signal groups.

**Figure E**


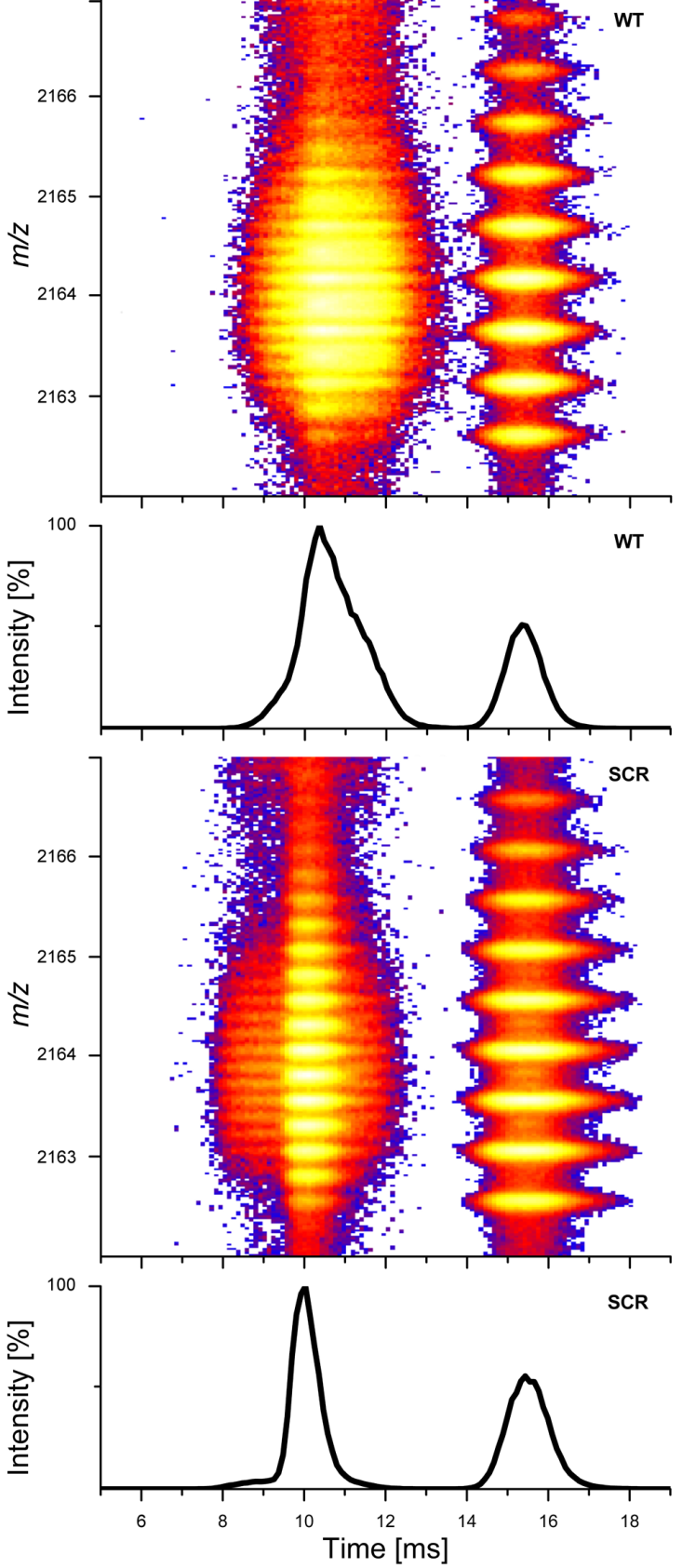


**Figure E.** A fragment of a two-dimensional IM-MS of WT Aβ 1-40 (upper panel) and SCR Aβ 1-40 (lower panel), covering *m/z* region 2163-2167 along with the corresponding drift time profile collected in negative ionization mode. In 2D rendering *m/z* values are shown at the vertical axis and ion mobility drift time at the horizontal axis. The signal amplitude is color-coded, increasing from pink to bright yellow. For WT two groups of signals can be resolved, a peak of longer drift time, corresponding to MON^2-^ and a peak of lower drift time broadened by the coalescence of DIM^4-^ and two alternative forms of TRI^6-^. For SCR the peak of smaller drift time is not broadened because of a weaker presence of trimeric signals, nevertheless well visible in 2D rendering. The same effect was observed for positive ionization mode (Fig 7C).

**Figure F**





**Figure F.** Circular dichroism spectra of WT Aβ 1-40 (line) and SCR Aβ 1-40 (dotted line), (i-iii) three batch-to-batch replicates.

**Figure G**


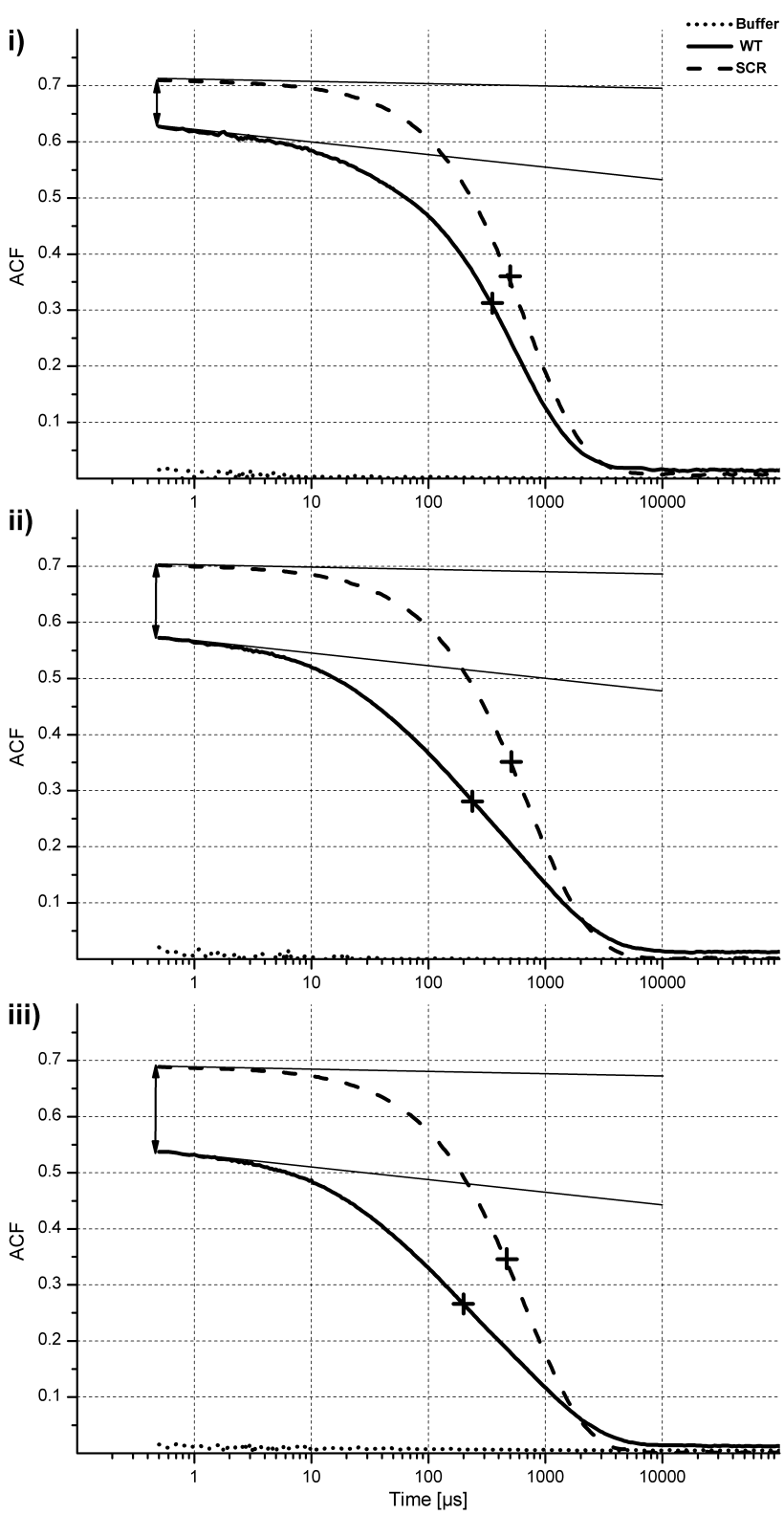


**Fig G.** DLS experiment of WT Aβ 1-40 and SCR Aβ 1-40. Representative autocorrelation function (ACF), directly adapted from dynamic light scattering data obtained for WT Aβ 1-40 (line) and SCR Aβ 1-40 (dashes). The autocorrelation function decays in the range of 1 down to 0, and the gap remaining to 1 observed at the shortest correlation time is indicative for the existence of monomeric or small oligomers fractions, which remain undetectable. The short-correlation-time asymptote of ACF is marked by thin solid lines. The mid-point of an ACF decay is marked by the crosses. Three batch-to-batch replicates (i-iii). It should be noted that autocorrelation time of 10 *μ*s roughly corresponds to objects of the mass 10 kDa (i.e. low-order Aβ oligomers), while autocorrelation time of 30 *μ*s is indicative for ~ 100 kDa oligomers (i.e. ~20-mers). Consequently, oligomers displaying in DLS larger autocorrelation times are undetectable in MS experiment.

**Figure H**


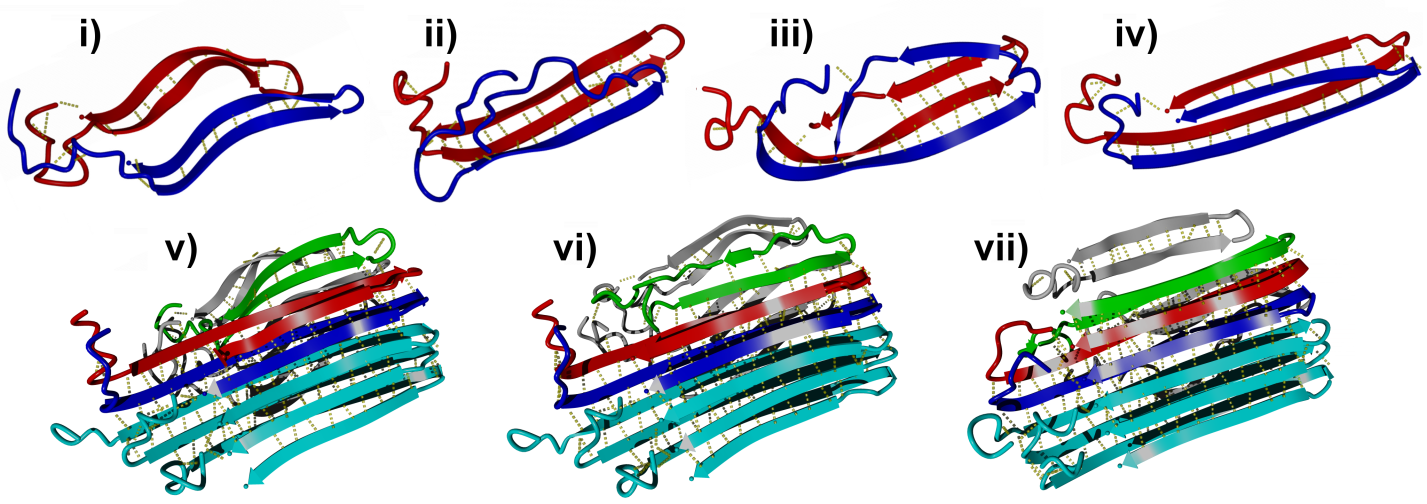


**Figure H.** Snapshots of the molecular dynamics trajectory visualizing a hypothetical pathway of the transition from a stacked β-hairpin oligomer (i) through nucleation of the parallel intermolecularly H-bonded dimer (ii-iv), which is next propagated across the whole oligomer (v-vii) towards the fully parallel β-structure oligomer. The directionality of strands is marked by arrow at the C-terminus. Yellow dotted lines indicate H-bonds, blue denotes the first chain converted, red the second and green the third, cyan marks parallel β-structured part while grey stacked antiparallel β-hairpins, next to be converted.

**Text A.** Description of the MD simulations. All MD simulations were performed with the aid of YASARA Structure package Ver.17.4.16 [24] using YASARA2 force field [25]. Simulations were run in the NTP ensemble with explicit water molecules, which initially filled the cube of the dimension of 100 Å. The whole system was initially equilibrated to obtain the mean solvent density of 0.997 g/cm^3^. Protonation state for each residue was set accordingly to pH of 7.5. All His residues were assumed neutral and arbitrary set in the Nᵟ1 protonation state. Several patterns of constrains were introduced to force system to enable switch between postulated topologies of interacting monomers. Backbone conformation of residues located in the β-sheet regions was forced by dihedral restraints (φ=-119ᵒ, ψ=113ᵒ, ∆=5ᵒ). Closure of individual backbone N-H…O hydrogen bonds were then controlled by the combination of distance restrains (d_NO_=2.9Å, ∆=0.2Å) and pseudo-dihedral restraints applied for C, N, Cα of the donor, and O of the acceptor (180ᵒ, ∆=5) to preserve the required geometry.

1. Hairpin-like antiparallel structure (Fig 9Ai) of the Aβ monomer was forced for residues His14-Val24, Gly29-Val39 introducing φ and ψ restraints together with the pattern of intramolecular hydrogen bonds identified in the X-ray structure of Aβ (PDB ID: 2OTK);
2. Mixed antiparallel/parallel Aβ oligomer units (Fig 9C) was obtained using hairpin-like constraints for one molecule together with a pattern of constraints for intermolecular H-bonding between His14-Val24 of the two paired parallel strands;
3. Open form of parallel sheet of two edge monomers of Aβ (Fig 9D) was obtained with the pattern of dihedral restraints applied for paired residues Gly14-Val24 and Gly29-Val39, combined with the expected pattern of hydrogen bonds formed between the two neighboring strands;
4. Closed Aβ sandwich-like structure of two units (Fig 9E) was stabilized by additional distance restraints between Cγ atoms of residues Leu17 and Leu34 of each molecule (dγγ=6.3Å, ∆=0.5Å);
5. Subsequent expansion of Aβ oligomers (Fig 9F,G) was further induced by combination of constraints corresponding to the closed form of parallel n-mer (4) extended by these for mixed antiparallel/parallel organization with the peripheral hairpin-like monomer (2).

Weights for constraints corresponding to a given type of oligomer topology varied upon simulation. In principle, the energy constants 10 kcal/mol/rad^2^ and 10 kcal/mol/Å^2^ were used for respectively dihedral and distance restrains to switch on the appropriate set of constraints, while all other ones were scaled to 0. The transition between two states was forced by resetting of all conflicting constrains to 0 and rescaling of the appropriate set of constraints in 5 successive steps (by 2 kcal/mol/rad^2^ and 2 kcal/mol/Å^2^, respectively), separated by 2ns of MD trajectory.

REFERENCES

1. Barghorn S, Nimmrich V, Striebinger A, Krantz C, Keller P, Janson B, et al. Globular amyloid beta-peptide oligomer - a homogenous and stable neuropathological protein in Alzheimer’s disease. J Neurochem. 2005;95: 834–47. doi:10.1111/j.1471-4159.2005.03407.x

2. Chimon S, Shaibat MA, Jones CR, Calero DC, Aizezi B, Ishii Y. Evidence of fibril-like β-sheet structures in a neurotoxic amyloid intermediate of Alzheimer’s β-amyloid. Nat Struct Mol Biol. 2007;14: 1157–64. doi:10.1038/nsmb1345

3. Hoyer W, Gronwall C, Jonsson A, Stahl S, Hard T. Stabilization of a β-hairpin in monomeric Alzheimer’s amyloid-β peptide inhibits amyloid formation. Proc Natl Acad Sci. 2008;105: 5099–5104. doi:10.1073/pnas.0711731105

4. Cerf E, Sarroukh R, Tamamizu-Kato S, Breydo L, Derclaye S, Dufrêne YF, et al. Antiparallel beta-sheet: a signature structure of the oligomeric amyloid beta-peptide. Biochem J. 2009;421: 415–23. doi:10.1042/BJ20090379

5. Ono K, Condron MM, Teplow DB. Structure-neurotoxicity relationships of amyloid beta-protein oligomers. Proc Natl Acad Sci U S A. 2009;106: 14745–50. doi:10.1073/pnas.0905127106

6. Yu L, Edalji R, Harlan JE, Holzman TF, Lopez AP, Labkovsky B, et al. Structural characterization of a soluble amyloid beta-peptide oligomer. Biochemistry. 2009;48: 1870–7. doi:10.1021/bi802046n

7. Zhang A, Qi W, Good TA, Fernandez EJ. Structural differences between Aβ(1-40) intermediate oligomers and fibrils elucidated by proteolytic fragmentation and hydrogen/deuterium exchange. Biophys J. Biophysical Society; 2009;96: 1091–104. doi:10.1016/j.bpj.2008.10.022

8. Yamaguchi T, Yagi H, Goto Y, Matsuzaki K, Hoshino M. A disulfide-linked amyloid-beta peptide dimer forms a protofibril-like oligomer through a distinct pathway from amyloid fibril formation. Biochemistry. 2010;49: 7100–7. doi:10.1021/bi100583x

9. Ahmed M, Davis J, Aucoin D, Sato T, Ahuja S, Aimoto S, et al. Structural conversion of neurotoxic amyloid-beta(1-42) oligomers to fibrils. Nat Struct Mol Biol. 2010;17: 561–7. doi:10.1038/nsmb.1799

10. Sandberg A, Luheshi LM, Söllvander S, Pereira de Barros T, Macao B, Knowles TPJ, et al. Stabilization of neurotoxic Alzheimer amyloid-beta oligomers by protein engineering. Proc Natl Acad Sci U S A. 2010;107: 15595–600. doi:10.1073/pnas.1001740107

11. Pan J, Han J, Borchers CH, Konermann L. Conformer-specific hydrogen exchange analysis of Aβ(1-42) oligomers by top-down electron capture dissociation mass spectrometry. Anal Chem. 2011;83: 5386–93. doi:10.1021/ac200906v

12. Lee J, Culyba EK, Powers ET, Kelly JW. Amyloid-β forms fibrils by nucleated conformational conversion of oligomers. Nat Chem Biol. 2011;7: 602–9. doi:10.1038/nchembio.624

13. Scheidt HA, Morgado I, Huster D. Solid-state NMR reveals a close structural relationship between amyloid-β protofibrils and oligomers. J Biol Chem. 2012;287: 22822–6. doi:10.1074/jbc.M112.367474

14. Stroud JC, Liu C, Teng PK, Eisenberg D. Toxic fibrillar oligomers of amyloid-β have cross-β structure. Proc Natl Acad Sci U S A. 2012;109: 7717–22. doi:10.1073/pnas.1203193109

15. Gu L, Liu C, Guo Z. Structural insights into Aβ42 oligomers using site-directed spin labeling. J Biol Chem. 2013;288: 18673–83. doi:10.1074/jbc.M113.457739

16. Huang D, Zimmerman MI, Martin PK, Nix AJ, Rosenberry TL, Paravastu AK. Antiparallel β-Sheet Structure within the C-Terminal Region of 42-Residue Alzheimer’s Amyloid-β Peptides When They Form 150-kDa Oligomers. J Mol Biol. 2015;427: 2319–28. doi:10.1016/j.jmb.2015.04.004

17. Tay WM, Huang D, Rosenberry TL, Paravastu AK. The Alzheimer’s amyloid-β(1-42) peptide forms off-pathway oligomers and fibrils that are distinguished structurally by intermolecular organization. J Mol Biol. 2013;425: 2494–508. doi:10.1016/j.jmb.2013.04.003

18. Lendel C, Bjerring M, Dubnovitsky A, Kelly RT, Filippov A, Antzutkin ON, et al. A hexameric peptide barrel as building block of amyloid-β protofibrils. Angew Chem Int Ed Engl. 2014;53: 12756–60. doi:10.1002/anie.201406357

19. Parthasarathy S, Inoue M, Xiao Y, Matsumura Y, Nabeshima Y, Hoshi M, et al. Structural Insight into an Alzheimer’s Brain-Derived Spherical Assembly of Amyloid β by Solid-State NMR. J Am Chem Soc. 2015;137: 6480–3. doi:10.1021/jacs.5b03373

20. Williams TL, Choi JK, Surewicz K, Surewicz WK. Soluble Prion Protein Binds Isolated Low Molecular Weight Amyloid-β Oligomers Causing Cytotoxicity Inhibition. ACS Chem Neurosci. 2015;6: 1972–80. doi:10.1021/acschemneuro.5b00229

21. Streltsov VA, Varghese JN, Masters CL, Nuttall SD. Crystal structure of the amyloid-β p3 fragment provides a model for oligomer formation in Alzheimer’s disease. J Neurosci. 2011;31: 1419–26. doi:10.1523/JNEUROSCI.4259-10.2011

22. Nisbet RM, Nuttall SD, Robert R, Caine JM, Dolezal O, Hattarki M, et al. Structural studies of the tethered N-terminus of the Alzheimer’s disease amyloid-β peptide. Proteins Struct Funct Bioinforma. Wiley-Blackwell; 2013;81: 1748–1758. doi:10.1002/prot.24312

23. Kłoniecki M, Jabłonowska A, Poznański J, Langridge J, Hughes C, Campuzano I, et al. Ion mobility separation coupled with MS detects two structural states of Alzheimer’s disease Aβ1-40 peptide oligomers. J Mol Biol. 2011;407: 110–24. doi:10.1016/j.jmb.2011.01.012

24. Krieger E, Vriend G. New ways to boost molecular dynamics simulations. J Comput Chem. Wiley-Blackwell; 2015;36: 996–1007. doi:10.1002/jcc.23899

25. Krieger E, Darden T, Nabuurs SB, Finkelstein A, Vriend G. Making optimal use of empirical energy functions: Force-field parameterization in crystal space. Proteins Struct Funct Bioinforma. Wiley-Blackwell; 2004;57: 678–683. doi:10.1002/prot.20251
